# Sex-specific benefits of a combined supplementation of B vitamins, nicotinamide riboside, folate and cobalamin, in a murine model of heart failure

**DOI:** 10.1186/s13293-025-00764-x

**Published:** 2025-10-21

**Authors:** Solène E. Boitard, Morgane Delouche, Ahmed Karoui, Mélanie Gressette, Iman Momken, Bertrand Bouchard, Françoise Mercier-Nomé, Apolline Imbard, Christophe Lemaire, Anne Garnier, Matthieu Ruiz, Mathias Mericskay, Jérôme Piquereau

**Affiliations:** 1 Signalling and Cardiovascular Pathophysiology, Inserm, UMR-S 1180, Université Paris-Saclay, Orsay, France; 2 Université d’Evry, Université Paris Saclay, Evry-Courcouronnes, France; 3 Montreal Heart Institute Research Centre, Montreal, Canada; 4 Department of Nutrition, Faculty of medicine, Université de Montréal, Montreal, Canada; 5 US31-UMS3679-Plateforme PHIC, Ingénierie et Plateformes au Service de l’Innovation Thérapeutique (IPSIT), INSERM, CNRS, Université Paris- Saclay, Orsay, France; 6 Department of Biochemistry, Assistance Publique-Hôpitaux de Paris, Paris, France; 7 Université Versailles St-Quentin, Université Paris-Saclay, Orsay, France; 8 Laboratoire PRéTI UR 24184, Université de Poitiers, Poitiers, France

## Abstract

Despite a substantial therapeutic arsenal to treat patients affected by heart failure (HF), no treatment specifically targets alterations of cardiac energy metabolism described in HF. Based on the results of previous studies demonstrating the cardiac preventive effects of B vitamins when introduced before inducing cardiac pressure overload in mice, we investigated the efficacy of a diet supplemented with a B vitamin cocktail (B3, B9 and B12 (3VitB)) to restore energy metabolism and improve cardiac function in an animal model of established HF. Four weeks after transverse aortic constriction (TAC) induction, male and female mice were treated with 3VitB. 3VitB increased life expectancy and reduced the TAC-induced alterations of cardiac parameters in males. Although these effects on survival and cardiac function were less clear in females due to their higher resistance to TAC, the 3VitB cocktail was beneficial in females as 8 weeks of treatment improved physical capacities and led to milder cardiomyocyte stress-induced hypertrophy in similar ways to those observed in males. In both sexes, 3VitB protected cardiac mitochondrial oxidative capacities, at least by supporting the process of mitochondrial biogenesis. Interestingly, our results revealed sex-specificities not only in response to cardiac pressure overload but also in response to 3VitB treatment. Overall, this study demonstrated the efficacy of 3VitB to preserved cardiac function and energy metabolism in an established HF model, especially in males that are more sensitive to cardiac pressure overload. This confers credit to vitamin supplementations and to metabolic therapy as new strategies to treat HF.

**Supplementary Information:**

The online version contains supplementary material available at 10.1186/s13293-025-00764-x.

## Highlights

- Alterations of energy metabolism and physical capacities are described in mouse model of established heart failure in males and females.
- Diet supplemented with nicotinamide riboside, vitamin B12 and B9 improved mitochondrial oxidative capacities and physical performance when introduced at an advance stage of heart failure.
- Males are more sensitive than females to the B vitamin cocktail treatment.
- Beneficial effects of these three B vitamins are associated with maintenance of mitochondrial biogenesis in both sexes.

**Supplementary Information:**

The online version contains supplementary material available at 10.1186/s13293-025-00764-x.

## Abstract (plain language summary)

Heart failure (HF) is a severe condition in which the heart struggles to pump blood effectively. While many treatments exist, none specifically target the changes in cardiac energy production processes (energy metabolism) that occur in this disease. In the present study, it has been explored whether a diet enriched with a cocktail of B vitamins (nicotinamide riboside (B3), folate (B9) and cobalamin (B12)), called 3VitB, could improve cardiac energy metabolism and function in a mouse model of established HF induced by cardiac pressure overload. Four weeks after cardiac stress induction, mice were treated with 3VitB. In male mice, this vitamin cocktail increased survival rates, improved heart function and physical capacities. Female mice, which naturally resist cardiac stress better, showed less marked improvements in survival and cardiac function, but after several weeks of treatment, they exhibited better physical performance and less harmful heart cell hypertrophy. In both sexes, 3VitB helped protect the heart’s mitochondria, the main energy producers of the cardiac cell, by supporting their renewal and function. The study also found differences between males and females in how their hearts responded to both stress and vitamin treatment. Overall, the findings of this study suggest that 3VitB supplementation helps preserve heart energy metabolism and function in HF, especially in males which are more vulnerable to cardiac stress. This supports the idea that vitamin supplements and metabolic therapies could be promising new approaches to treat HF.

**Supplementary Information:**

The online version contains supplementary material available at 10.1186/s13293-025-00764-x.

## Introduction

Despite constant advances in medical science, prevalence of heart failure (HF) is still high and is expected to rise in the world with ageing of the population and the spread of western diet and associated comorbidities. This syndrome, which is often the terminal stage of diverse chronic cardiovascular diseases (CVD), is a major cause of death worldwide and cannot be fully cured in spite of important efforts to improve therapeutics these last decades.

It is now well-established that profound modulations of energy metabolism are involved in the development of HF. The failing heart is in an energy-depleted state [1], which is associated with a shift from predominant fatty-acid oxidation to an increase in ketone bodies and glucose uptake, although for the latter its use remains confined to glycolysis and ancillary pathways and disconnected of further oxidation due to the severe mitochondrial dysfunction occurring in HF [1, 2]. While the underlying reasons for such alterations remain only partially understood, the knowledge acquired from HF animal models and HF patients on the dysregulations of mitochondrial life cycle [3] and the disruption of nicotinamide adenine dinucleotide (NAD+) homeostasis [4, 5] confers credit to the concept of a metabolic therapy that would aim to restore the energy metabolism of the failing heart. For decades, none of the main drugs in the therapeutic arsenal currently used to treat HF in clinical practice, β-blockers and angiotensin converting enzyme 2 inhibitors inhibitor, specifically addressed the issue of altered cardiac energy metabolism. More recently, the use of sodium-glucose-cotransporter 2 inhibitors or glucagon-like peptide-1 receptor agonists that were originally designed as first-line therapies for diabetes but also emerged as novel treatment options for HF therapy, revived the idea that modulators of energy metabolism could be of the greatest medical significance for the treatment of this syndrome. These new clinical findings prompted the research community to pursue the metabolic strategy and design strategies to stimulate cardiac energy metabolism.

Micronutrient deficiency has long been known as one of the feature characterizing patients with HF, particularly those with advanced disease who are prone to cachexia and malnutrition due to multiple causes [6, 7]. Among micronutrient B vitamins are essential precursors of the cofactors required for mitochondrial enzymes involved in energy metabolism [8]. Vitamin supplementation has so far mostly been assessed as a prophylactic measure to reduce the risk of developing cardiovascular disease. These studies, many of them underpowered, showed modest benefits at best for cardiovascular diseases and even adverse effects for some like vitamin E increasing cancer risk [9]. However, the rationale of this approach is questionable as the studied populations may simply not be in a state of micronutrient deficiency in the heart as long as they do not reach a stage of advanced HF.

What has not been assessed so far is whether supplementation in some of those B vitamins could actually slow down the progression of established HF and modify the outcome by restoring mitochondrial function.

In this context, our team has previously demonstrated the therapeutic capacity of B vitamins in mouse models of HF [10, 11]. Our first study [10] showed that the protective effects of cobalamin (B12) and folate (B9) that impact on the homocysteine/methionine cycle preserves left ventricular function and mitochondrial oxidative capacity in a mouse model of pressure overload triggered by transverse aorta constriction (TAC). We also showed that nicotinamide riboside (NR), a nucleoside form of the vitamin B3 that functions as a precursor for the nicotinamide adenine dinucleotide (NAD) cofactor, is also beneficial to reduce cardiac dilatation and improves outcome in a model of non-ischemic dilated cardiomyopathy as well as in the TAC model [11] and as also shown by others [12–14].

Beside their role in mitochondrial metabolism, vitamins B3, B9 and B12 also have signaling function as precursors for NAD^+^-mediated deacetylation by sirtuins family members and for S-adenosine methionine (SAM), the methyl donor used by methyl transferases [8]. They can thus converge on the activation of the peroxisome proliferator-activated receptor gamma co-activator 1 α (PGC-1α), a master regulator of energy metabolism and mitochondrial life cycle, by promoting its methylation by protein arginine methyltransferase 1 (PRMT1) and its deacetylation by sirtuin-1 (SIRT1), two activating post-translational modulations of this co-activator [15, 16].

SIRT1 is a deacetylase that regulates many facets of energy metabolism and its central role makes it a prime target for the development of metabolic therapies of HF. However, its activity requires NAD^+^ [17] and the loss of myocardial NAD^+^ in HF [4] could be a hindrance to the development of such a therapy. Indeed, this lack of NAD^+^ could limit the effect of SIRT1 activation strategies on the one hand and on the other hand fully efficient catabolism requires NAD^+^ at many stages, especially for mitochondrial functions [18]; this implies that suitable NAD^+^ level would be a prerequisite for any effective metabolic therapy. In an interesting way, in our second aforementioned study [11] our team showed that treatment with NR restores cardiac NAD^+^ level and protects cardiac function in mouse models of dilated cardiomyopathy or TAC induced-pressure overload. According to our analysis, in these models NR would take advantage of an increase in the expression of the NMRK2 enzyme that catalyzes the synthesis of nicotinamide mononucleotide (NMN) (the immediate upstream precursor of NAD^+^) from NR, thereby compensating for the decrease in nicotinamide phosphoribosyltransferase (NAMPT) activity which ensures NAD^+^ production from nicotinamide (NAM) in the healthy heart [19].

Based on these encouraging results, we chose to assess in the present study the effect of a vitamin cocktail containing NR, folate and cobalamin in a murine model of pressure overload-induced HF. While the effects of these three B vitamins were assessed by preventive administration at early stage before the onset of the first HF symptoms in our previous studies, here we decided to assess the efficacy of the vitamin B3-9-12 cocktail (3VitB) in a model of established HF to reveal its potential effects at a more advanced stage of the disease, as would be the case with HF patients. In addition, considering the key importance of studying the potential differences in response to treatments between males and females in preclinical research to improve translational potential of our finding, we decided to include males and females separately in our study, especially because until now females had not been assessed in our preclinical studies with these vitamins.

## Materials and methods

### Animals

All animal experimental procedures were approved by animal ethics committee of Paris-Sud University, authorized by French government (authorization number: APAFIS#24778-2020032416337862) and complied with directive 2010/63/EU of the European Parliament on the protection of animals used for scientific purposes. Heart failure was induced in male and female C57BL/6NCrl mice (Charles River Laboratories, breeding site Italy) subjected to pressure overload by surgical transverse aortic constriction (TAC) performed according to surgery procedure described in supplementary information.

### Treatment administration on non-synthetic diet

Four weeks after surgical transverse aortic constriction (TAC), mice were randomized based on different inclusion criteria assessed by trans-thoracic echocardiography: pressure gradient above 60mmHg (Figure S2B), decrease of 10% of left ventricular ejection fraction (LVEF) and increase of 30% of left ventricular mass (Figure S2C). Mice that did not match two or more of these criteria were excluded from the study. After randomization, male and female mice were divided into three groups: (1) sham-operated mice receiving standard SAFE^®^ A04 non-synthetic diet (SAFE, Augy, France), (2) TAC mice receiving standard SAFE^®^ A04 non-synthetic diet called TAC-ND, (3) TAC mice receiving customized SAFE^®^ A04 supplemented with NR (4 g/kg), cobalamin (1 mg/kg) and folate (20 mg/kg) called TAC-3VitB in this manuscript. Animals were housed under temperature-controlled conditions (21 °C) and had free access to water and to their respective food. Supplementation with vitamins started four weeks after TAC surgery and two different treatment times were evaluated in two batches (Figure S1A and B): (1) 20 weeks of vitamins treatment for survival and cardiac function study in males (Sham *n* = 11; TAC-ND *n* = 20; TAC-Vit *n* = 19) and females (Sham *n* = 11; TAC-ND *n* = 16; TAC-Vit *n* = 20); (2) 8 weeks of vitamins treatment for studying physical capacities, metabolomic, histologic, and molecular analysis in males (Sham *n* = 9; TAC-ND *n* = 11; TAC-Vit *n* = 11) and females (Sham *n* = 6; TAC-ND; *n* = 10; TAC-Vit *n* = 10).

### Survival

A survival study was carried out during 20 weeks of treatment to evaluate the impact of treatment on lifetime in males and females. A regular monitoring of body weight (each week) and cardiac function by trans-thoracic echocardiography (every four weeks) were done and correlated to end points determined on ethical project. When mice lost more than 20% of total body weight, had a left ventricular ejection fraction under 20% or reached end points, mice were euthanized and marked on survival curve as an event.

### Effort test on treadmill

Before introduction and after 8 weeks of treatment, the ability of the heart to adapt to physical effort was evaluated by an exertion test on treadmill (Ugo Basile, France) in male and female mice. Three days before the effort test, an adaptation phase was carried out with the following program: three and two days before the test, the mice were subjected to the following training session during which the treadmill started at a 3 m.min^−1^ speed before being increased by 1 m.min^−1^ every 2 min for 10 min without slope. After a day off, the exhaustion test was performed with the following program: after an acclimation of 30 min, the treadmill started at a 3 m.min^−1^ speed that was increased by 3 m.min^−1^ every 3 min without slope to determine the maximal running time under increasing speed.

### Echocardiography

Every four weeks, cardiac function was evaluated by trans-thoracic echocardiography with a Vevo 3100 device (FUJIFILM Visualsonics Inc., Toronto, Canada) equipped with a linear array 22–55 MHz MicroScan mouse cardiovascular transducer (MS550). Echocardiographic measures were performed on anesthetized mice using isoflurane (Centravet, Plancoet, France), 3% for induction and 1 to 2% for the maintenance of anesthesia (see supplementary information for further details). Images were analyzed using VevoLab Software (FUJIFILM Visualsonics Inc., Toronto, Canada).

### Histological analysis

Hearts were fixed in 4% paraformaldehyde, paraffin embedded and serially sectioned (3 μm).

Sections were stained with Sirius red (Ab150681; Abcam) for studying fibrosis or with FITC-labeled wheat germ agglutinin (WGA) to label cell membranes. The slides were scanned using NanoZoomer2.0-RS^®^ (Hamamatsu Photonics) digital scanner. Using Image J software, fibrosis and cross-sectional area of the fibers were quantified from 12 to 15 fields of a transversal section of the left ventricle for each animal.

### Mitochondrial oxidative capacities assays

Mitochondrial respiration was studied in situ in saponin-permeabilized cardiac muscle fibers using a Clarke electrode (O2k-Fluorespirometer, Oroboros Instruments) as previously described [20]. Preparation of permeabilized fibers is detailed in supplementary information. Oxygen consumption of the fibers were measured after successive addition of pyruvate 1 mM, malate (4 mM), ADP (2 mM), succinate (15 mM), amytal (an inhibitor of complex I, 1 mM) and N, N, N’,N’-tetramethyl-p-phenylenediamine dihydrochloride (TMPD)-ascorbate (0.9:9mM) to respiration solution. Rates of respiration are given in nmoles O2/min/mg dry weight. The respiratory acceptor control ratio (ACR) was calculated from basal mitochondrial respiration rate (in the presence of pyruvate/malate 1/4 mM, without ADP) and oxygen consumption after addition of 2mM ADP.

### Enzyme activity

Frozen tissue samples were weighed, homogenized (Bertin Precellys 24) in ice-cold buffer (50 mg/ml) containing 4-(2-hydroxyethyl)−1-piperazineethanesulfonic acid (HEPES) 5 mM (pH 8.7), EGTA 1 mM, DTT 1mM and 0.1% Triton X-100. Enzymatic activities were measured spectrophotometrically using standard coupled enzymes assays at 30°C. Citrate synthase (CS) activity was measured in a Tris-HCl buffer (pH 8) containing acetyl-Coa (10 mM), oxaloacetic acid (10 mM) and 5,5’-dittiobis-(2-Nitrobenzenoic acid) (DTNB) that allows, through coupled reactions involving CS, the production of the C_6_O_4_S_2_^−^ ion the apparition of which was followed at 412 nm. Complex I (NADH-CoQ reductase) activity was determined by monitoring the disappearance of NADH at 340 nm in a 50 mM phosphate buffer (KH_2_PO_4_ (0.411 mM), K_2_HPO_4_ (0.088 mM), pH 7.4) containing 3.75 mg/ml BSA, 100 µM NADH and 100 µM of decyl-ubiquinone that played the role of electron acceptor. Results are given in IU/g protein.

### Quantitative Real-time PCR

Frozen tissue samples were weighed and homogenized (Bertin Precellys 24) in Trizol reagent (Invitrogen) allowing total ventricular RNA extraction. After addition of chloroform, samples were centrifugated at 10.000 g at 4 °C to obtain three phases: phenol-chloroform fraction, interphase and aqueous phase containing proteins, DNA and RNA respectively. Total RNAs were then precipitated by the addition of isopropanol to the aqueous phase, centrifugated at 10.000 g and resuspended in DNase/RNase free water. Oligo-dT first strand cDNA was synthesized from 1 µg of total RNAs using iScript cDNA synthesis kit (Bio-rad). Negative controls without reverse transcriptase enzyme were performed to verify the absence of genomic DNA in the samples. Real-time PCR was performed using the SYBR^®^ green method on CFX96 TouchTM Real Time PCR Detection system (Bio-Rad) from 2.5 ng cDNA. mRNA levels for all target genes were normalized to Tyrosine 3-Monooxygenase/Tryptophan 5-Monooxygenase Activation Protein Zeta (YWHAZ) expression level. Primer sequences are available in Table S1.

### Vitamin B12 dosage

Frozen myocardium tissue samples were weighed and homogenized in HCl (1 N). Two-step immunoassay for the quantitative determination of vitamin B12 using chemiluminescence microparticle immunoassay (CMIA) on Alinity i ^®^ multiparametric instrument (Abbott, IL, USA) was performed.

### Liquid Chromatography-Mass spectrometry

A liquid-chromatography coupled to mass spectrometry analysis was developed and carried out to quantified Methyl-NAM. This procedure is detailed in supplementary information.

### Statistical analysis

The results are presented as the means ± standard error of the mean (SEM), with individual values for each condition. Statistical analysis was performed using GraphPad Prism 9 software (GraphPad Software, RITME, France). The significance of the differences between groups was evaluated first after verifying the normality of the distribution by a Shapiro-Wilk normality test. If the distribution was normal, an ordinary one-way analysis of variance (ANOVA) followed by Tukey’s multiple comparisons test was done. If it was not normal, a nonparametric Kruskal-Wallis test followed by an uncorrected Dunn’s test was chosen. To compare significance on two parameters such as treatment and sex, two-way analysis of variance (ANOVA) followed by uncorrected Fisher’s LSD test was done. P values ≤ 0.05 were considered statistically significant. For the survival curve, to compare two groups, a Logrank (Mantel-Cox test) analysis was done.

## Results

### 1. The 3VitB cocktail improves survival and physical capacities of mice subjected to cardiac pressure overload

**Fig. 1:**
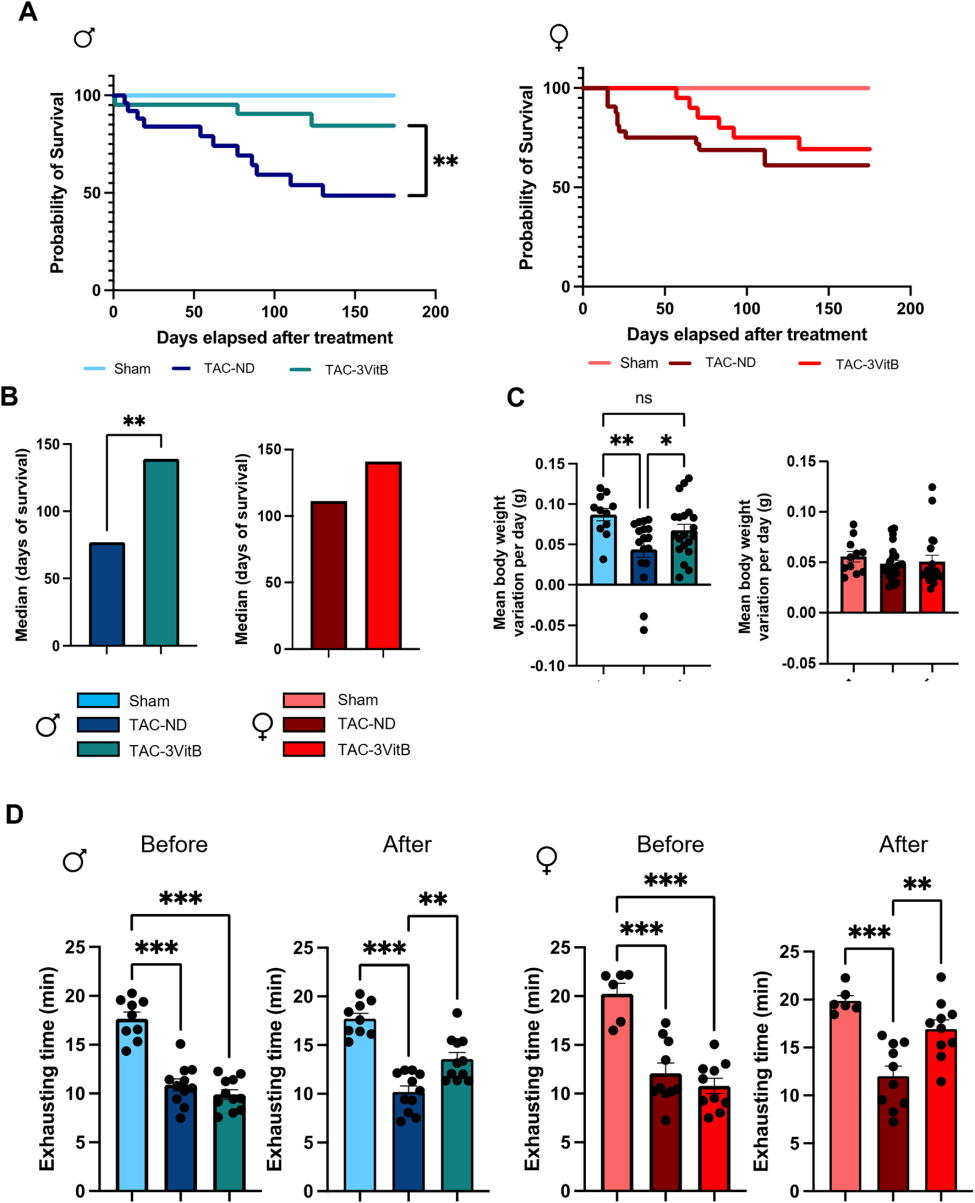
The 3VitB cocktail improves survival and physical capacities of mice subjected to cardiac pressure overload. (**A**) Survival curve analysis of males and females treated with ND or 3VitB compared using log-rank test. (**B**) Median survival after diet introduction. (**C**) Body weight gain from surgery to death or to sacrifice 24 weeks after surgery. (**D**) Maximal of running time on increasing speed on treadmill before and 8 weeks after treatment introduction. (n = 11 to 20), ** p < 0.01, *** p < 0.001

Cardiac pressure overload induced mortality over time in mice fed with non-supplemented diet (ND) or diet supplemented with 3VitB cocktail when compared with sham groups in both sexes (Fig. 1A). Survival was significantly improved in males fed with 3VitB as shown by Kaplan-Meier curve and the significant increase in median survival (Fig. 1B). These effects were combined with a beneficial impact on the normal body weight gain in males with ageing that was impaired in male TAC-ND group (Fig. 1C). Females also showed a trend towards a lower mortality rate and a higher median survival in TAC-3VitB group in comparison with TAC-ND group, although it remained below study’s defined significance level (Fig. 1A and B). This difference might be explained by the higher median survival in TAC-ND group in females than in males (Fig. 1B and supplementary table S3, *p* = 0.03). Also of note, TAC did not affect weight gain in females suggesting they resist better to the cardiac injury caused by TAC (Fig. 1C).

Exercise stress testing is an important noninvasive test to diagnose and risk stratify HF in patients. Although analysis of exercise capacities before treatment at 4 weeks after TAC showed an equal reduction in the animals randomly assigned to ND and 3VitB treatment, in females as well as in males, exercise capacities were significantly improved by 8 weeks of 3VitB treatment in both sexes (Fig. 1D) and females showed higher capacities than males (Table S3, *p* = 0.003).

### 2. Cardiac function of mice affected by HF is protected by the 3VitB cocktail, especially in males

Since physical capacities are used by New York Heart Association to stratify the stage of HF, the improved exercise capacities on treadmill in TAC-3VitB groups could suggest a better preservation of cardiac function after 3VitB treatment introduction in this model. Importantly, we verified and confirmed that the TAC procedure triggered the same level of constriction and pressure gradient across the aortic cross in males and females before treatment as assessed by pulse wave Doppler analysis (Supplementary Figure S2A and B and Table S3, *p* > 0.99), resulting in similar increase in LV mass and reduction in LVEF in both sexes at 4 weeks before treatment (Supplementary Figure S2C), ruling out any differences in early TAC response as a cause of subsequent evolution in cardiac function.

**Fig. 2:**
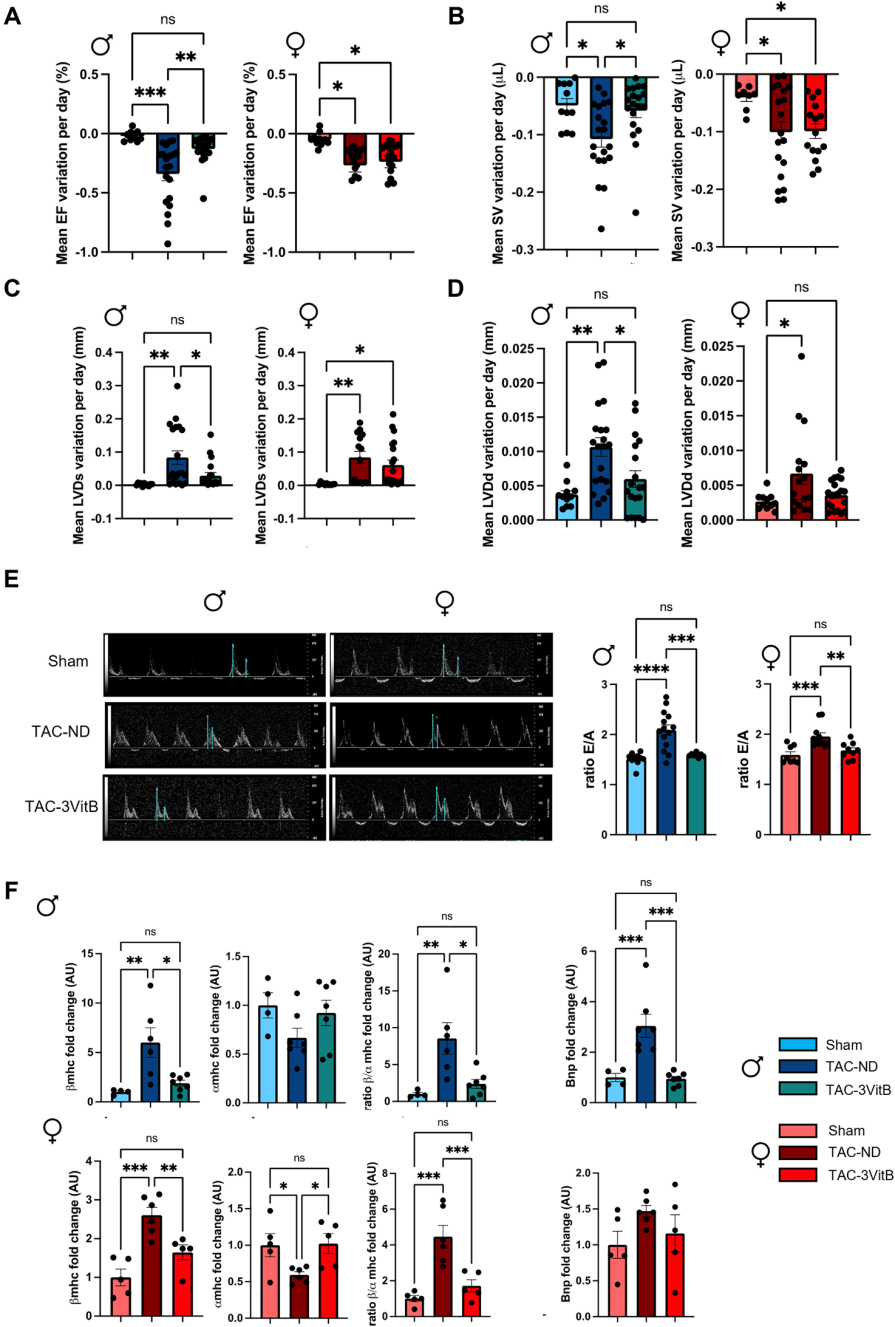
Cardiac protective effects of the 3VitB cocktail. A. Ejection fraction (EF) progression from surgery to death or to sacrifice 24 weeks after surgery. **B.** Stroke volume (SV) progression from surgery to death or to sacrifice 24 weeks after surgery **C.** End-systolic left ventricle diameter (LVDs) progression from surgery to death or to sacrifice 24 weeks after surgery. **D**. End-diastolic left ventricle diameter (LVDd) progression from surgery to death or to sacrifice 24 weeks after surgery **(E)** Representative echocardiographs and E wave/A wave ratio (E/A ratio) 8 weeks after treatment introduction **(F)** mRNA expression level of β-myosin heavy chain (*β-Mhc*), α-myosin heavy chain (*α-Mhc*) and brain natriuretic peptide (*Bnp*). (*n* = 11 to 20), * *p* < 0.05, ** *p* < 0.01, *** *p* < 0.001

In male TAC-3VitB group, the degradation of left ventricular ejection fraction (LVEF), stroke volume (SV) and the increase of LV end-systolic (LVDs) and -diastolic diameters (LVDd) over the time course of the survival study, were slower than in TAC-ND group (Fig. 2A-D), thereby confirming a benefit of these vitamins on the preservation of LV function and remodeling. In females, LVEF, SV and LVDs were similarly affected in TAC-ND group as compared to males (Fig. 2A-C and Table S3, *p* > 0.05 between sexes in TAC-ND groups) although LVDd was less increased (Fig. 2D, Table S3, *p* = 0.01 between males TAC-ND and females TAC-ND). The 3VitB treatment did not improved these parameters in females. Interestingly, the evaluation of the late diastolic transmitral flow velocity (E/A ratio) showed a similar increase 8 weeks after TAC in males and females (Fig. 2E and Table S3, *p* = 0.17 between sexes in TAC-ND groups) indicative of LV diastolic dysfunction and 3VitB treatment reduced this ratio to normal levels in both sexes.

The quantification in LV myocardium of transcripts of molecular markers of HF in mice showed the characteristic shift from cardiac fast α-myosin heavy chain (*Mhc*) to slow *βMhc* and the increase in brain natriuretic peptide (*Bnp*) gene expression associated with LV dilation in males TAC-ND group; and the 3VitB treatment robustly reduced these markers (Fig. 2F). In females, the *βMhc*/*αMhc* ratio increased less after TAC than in males (Fig. 2F and Table S3, *p* = 0.02 between sexes in TAC-ND groups) and it was rescued by the 3VitB treatment. *Bnp* expression was much less induced by TAC in females in agreement with the milder dilation of the LV chamber.

### 3. The 3VitB cocktail prevents the development of cardiac hypertrophy and fibrosis induced by cardiac pressure overload

**Fig. 3:**
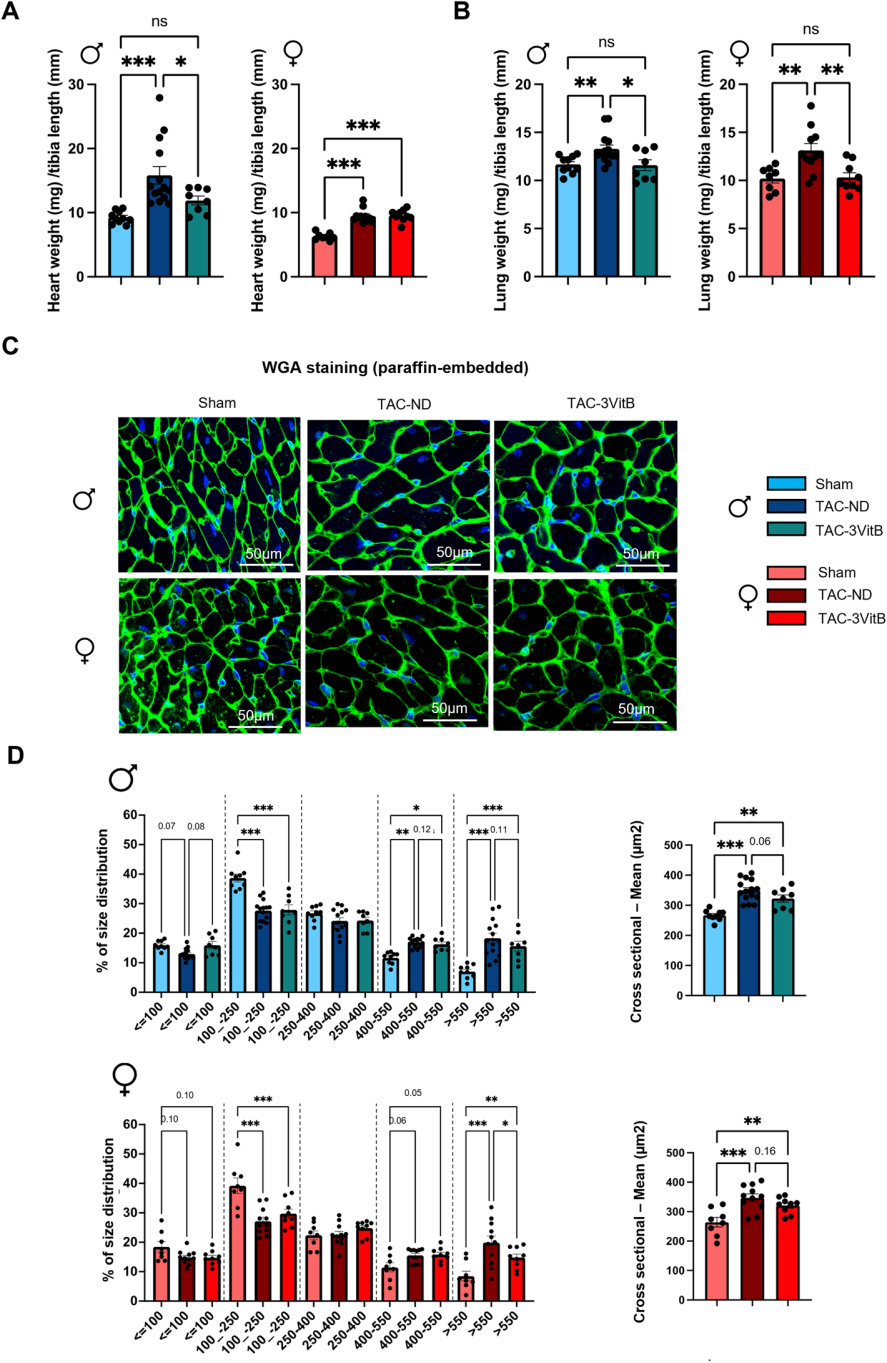
The 3VitB cocktail prevents the development of cardiac hypertrophy. **(A)** Heart weight to tibia length ratio (HWTL) at sacrifice 8 weeks after 3VitB treatment introduction. **(B)** Lung weight to tibia length ratio (LXTL) at sacrifice 8 weeks after 3VitB treatment introduction. **(C)** Representative pictures of cardiomyocyte hypertrophy analysis by fluorescein isothiocyanate (FITC)-labeled wheat germ agglutinin (WGA) after 8 weeks of 3VitB treatment. **(D)** proportion of cardiomyocytes by cross sectional size category, mean cross sectional area. (*n* = 6 to 8 animals per group), * *p* < 0.05, ** *p* < 0.01, *** *p* < 0.001

**Fig. 4:**
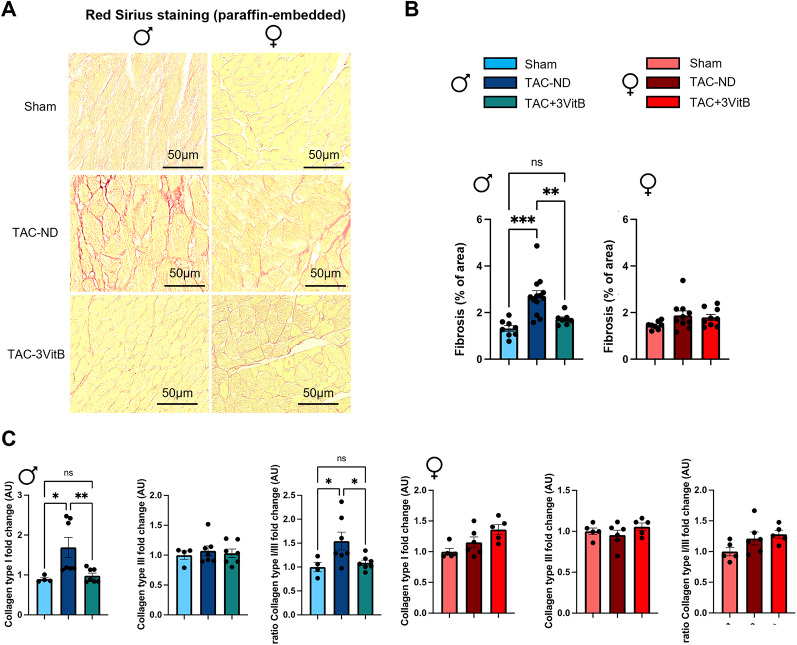
Myocardial fibrosis after 8 weeks of treatment. **(A)** Representative pictures of myocardial fibrosis analysis by Sirius red after 8 weeks of 3VitB treatment. **(B)** Proportion of total fibrosis in the different experimental groups at sacrifice 8 weeks after 3VitB treatment introduction. **(C)** Left ventricular mRNA expression level of collagen I (*Col1a*) and collagen III (*Col3a*). (*n* = 6 to 8 animals per group), * *p* < 0.05, ** *p* < 0.01, *** *p* < 0.001

In order to study the potential effects of the 3VitB cocktail on cardiac structure, a series of animals was especially designed to be sacrificed after 8 weeks of B vitamins supplementation, i.e. 12 weeks after TAC (Figure S1B). In males, heart weight-to-tibia length ratio (HWTL) at sacrifice was significantly increased in TAC-ND group and reduced in the TAC-3VitB group (Fig. 3A). In females, HWTL was significantly increased in TAC-ND group but much less than in males and the 3VitB treatment did not further reduced this parameter (Fig. 3A, and Table S3, *p* < 0.001 *p* = 0.02 between sexes in TAC-ND groups). In contrast, TAC increased lung weight-to-tibia length ratio in both sexes suggesting similar levels of pulmonary oedema and the 3VitB had same beneficial impact in both sexes (Fig. 3B). In spite of the lower increase of HWTL ratio triggered by TAC in females compared to males, the mean cross-sectional area of cardiac fibers and the proportion of very thick cardiomyocytes showed similar and significant increase in both sexes in TAC-ND groups (Fig. 3C and D). This confirmed the activation of the hypertrophy process in these groups that was counterbalanced by 3VitB treatment as judged by the “treatment effect” revealed by the 2-way ANOVA statistical analysis including males and females together (Table S3, treatment factor *p* < 0.001). This histological analysis was completed with Sirius red staining that showed a significant rise in cardiac fibrosis in TAC-ND group in males that was prevented by the 3VitB supplementation (Fig. 4A and B). This observation is reinforced by the measurement of the expression of collagen I that showed a significant increase in the TAC group fed with ND (Fig. 4C) while no increase was reported when mice were under 3VitB treatment. No expression change in collagen III was measured in TAC groups. Remarkably, no such increase in cardiac fibrosis and collagen I expression was observed in the female TAC-ND group (Fig. 4A, B and C).

### 4. Energy metabolism of mice affected by HF is preserved by the 3VitB cocktail, especially in males

**Fig. 5:**
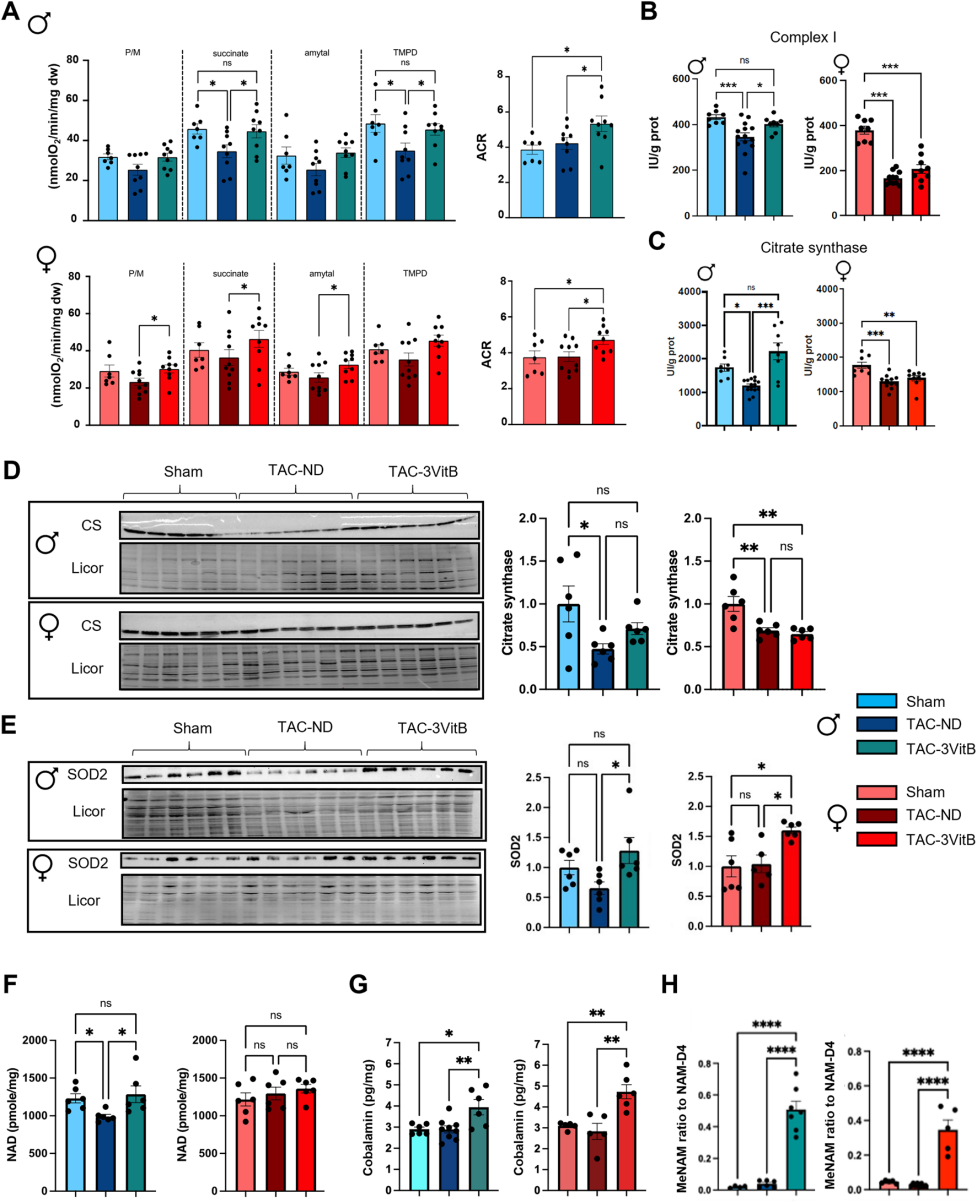
Cardiac mitochondrial oxidative capacities of mice affected by HF is preserved by 8 weeks of 3VitB cocktail treatment. (**A**) Rate of respiration measured under phosphorylating conditions (2mM ADP) in the presence of substrates for complex I (P/M), complexes I + II (succinate), complex II (Amytal) and complex IV (TMPD). (**B**) Complex I enzymatic activity. (**C**) Citrate synthase (CS) enzymatic activity. (**D**) Citrate synthase (CS) protein level. (**E**) SOD2 protein level. (**F**) Myocardial NAD level. (**G**) Myocardial cobalamin level. (**H**) Myocardial Methyl-NAM (MeNAM) level. (*n* = 6 to 8 animals per group), * *p* < 0.05, ** *p* < 0.01, *** *p* < 0.001

Considering the major role of B vitamins in energy metabolism and mitochondrial metabolism in particular, key elements of energy metabolism were characterized in the myocardium of the animals sacrificed 8 weeks after the beginning of the treatment. In males, mitochondrial oxidative capacities measured in the presence of substrates feeding complex I and II and direct activation of complex IV were altered in the TAC-ND group and fully rescued by the 3VitB treatment (Fig. 5A). In females, mitochondrial respiration rates were only mildly affected in the TAC-ND group, although they were not significantly different than in TAC-ND males (Table S3). Yet they were significantly increased by the 3VitB treatment.

Interestingly, the beneficial impact of the 3VitB treatment on mitochondrial oxidative capacities was also revealed by the higher respiratory acceptor control ratio (ACR) as measured in NAD-dependent complex I driven respiration in both males and females in comparison with Sham and TAC-ND groups (Fig. 5A). The decrease in mitochondrial oxidative capacities in TAC-ND males was associated with significant reduction of complex I biochemical activity (Fig. 5B) and citrate synthase (CS) activity and protein level (Fig. 5C and D), CS being usually used as a marker of mitochondrial mass; these alterations were prevented by 3VitB supplementation. In females, despite their lesser sensitivity to TAC, the activities of these enzymes and CS protein level were affected in the TAC-ND group (Fig. 5B, C and D) and not restored by 3VitB cocktail. The measurement of the mitochondrial superoxide dismutase 2 (SOD2) protein level revealed a rise in protein level in both TAC-3VitB groups that suggests that the 3VitB treatment could promote an antioxidant system (Fig. 5E). Higher oxidative capacities in TAC-3VitB males were associated with prevention of NAD level drop induced by TAC (Fig. 5F). Incidentally, the assessment of NAD and cobalamin level in myocardium showed significant increases in TAC-3VitB groups that demonstrated the assimilation of NR and vitamin B12 by the 3VitB treated mice (Fig. 5F and G). Methyl-NAM metabolite that represents the end-pathway product of both vitamin B3 elimination (nicotinamide) and vitamins B12/9 action on methylation, was induced by the 3VitB diet in both sexes (Fig. 5H).

### 5. The 3VitB cocktail sustained mitochondrial biogenesis transcription cascade in pressure overloaded myocardium in males and females

**Fig. 6:**
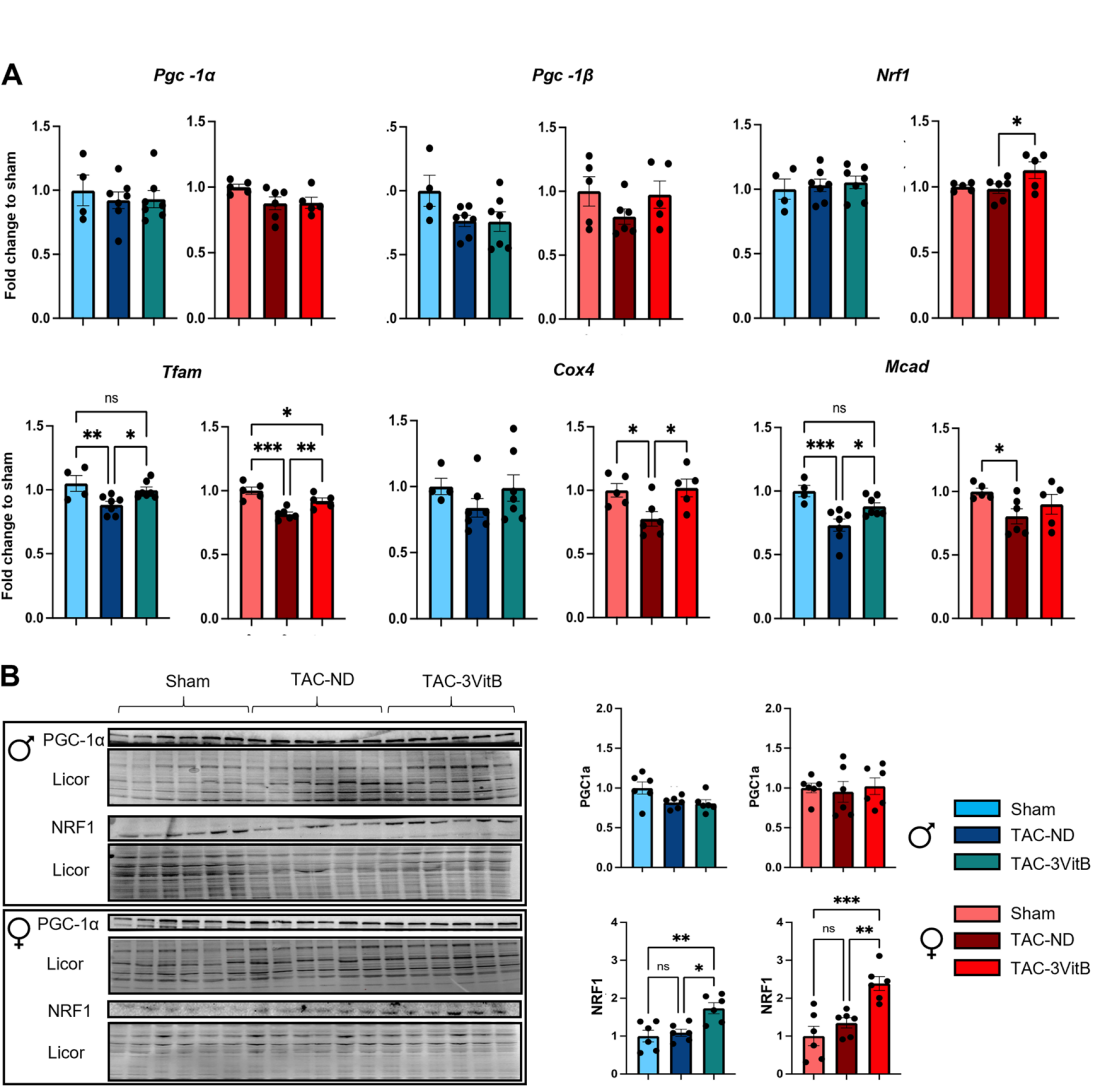
Cardiac mitochondrial oxidative capacities of mice affected by HF is preserved by 8 weeks of 3VitB cocktail treatment. (A) mRNA expression level peroxisome proliferator-activated receptor gamma co-activator 1 α (*Pgc-1α*), peroxisome proliferator-activated receptor gamma co-activator 1 β (*Pgc-1β*), nuclear respiratory factor 1 (*Nrf1*), mitochondrial transcription factor A (*Tfam*), cytochrome oxidase subunit 4 (Cox4) and the medium-chain acyl-CoA dehydrogenase (*Mcad*). (B) PGC-1α and NRF1 protein level

Although alterations and preservation of oxidative capacities were observed in TAC-ND and TAC-3VitB males respectively, no significant modulation of *Pgc-1α*, *Pgc-1β* and *Nrf1* transcripts level was noticed (Fig. 6A). The expression of these three genes involved in mitochondrial biogenesis was not altered in TAC-ND females neither when compared to sham group and only a slight increase in *Nrf1* expression was noticed in TAC-3VitB female group in comparison with Sham and TAC-ND groups. The 3VitB cocktail globally normalized the expression of mitochondrial transcription factor A (*Tfam*), cytochrome c oxidase 4 (*Cox4*) and medium-chain acyl-CoA dehydrogenase (*Mcad*) in both sexes, with some minor differences (Fig. 6A). At the protein level, PGC-1α protein level was not altered by surgery or treatment but a significant increase in NRF1 protein level was observed in TAC-3VitB groups in both males and females (Fig. 6B).

**Fig. 7:**
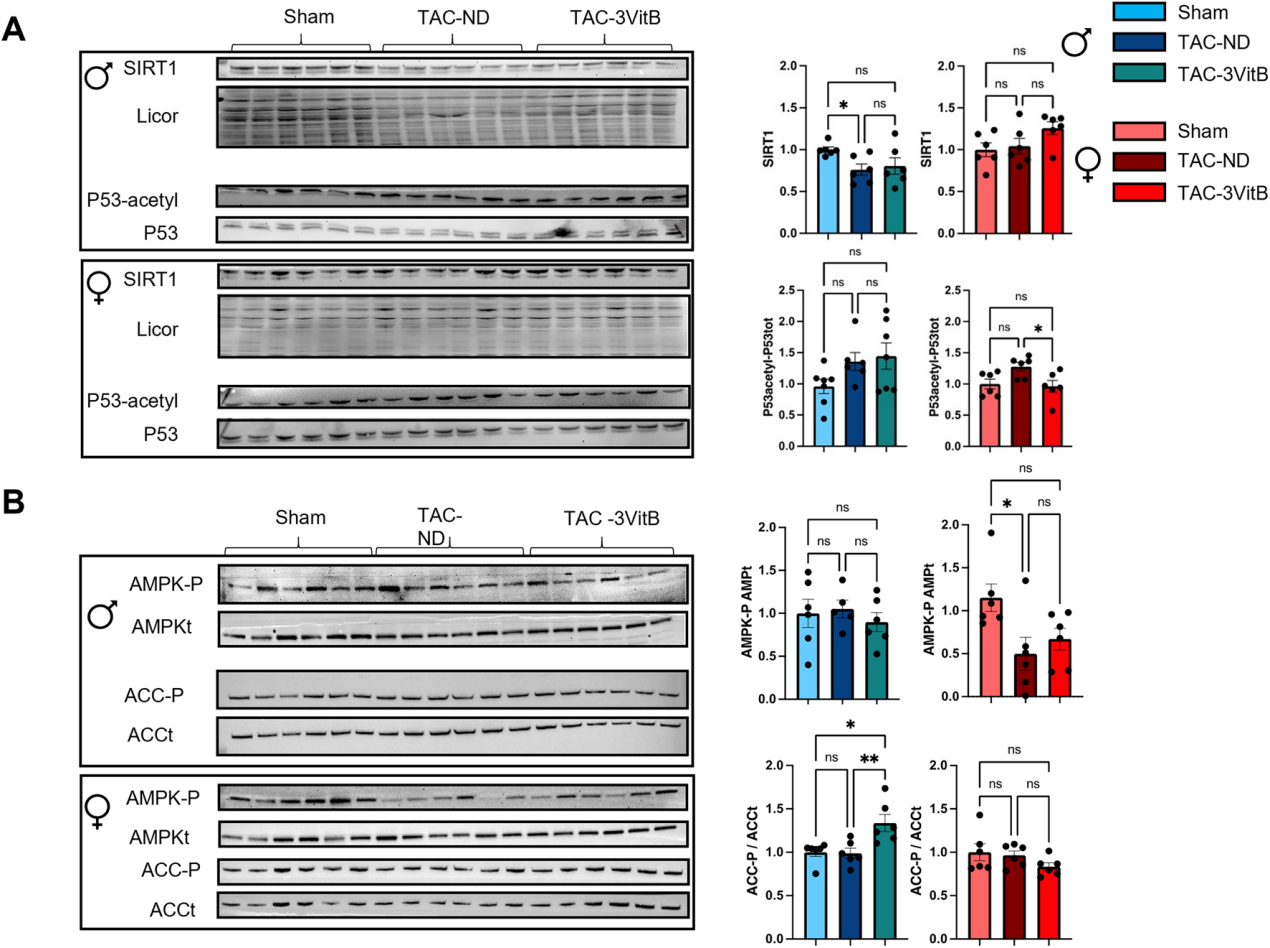
Cardiac mitochondrial oxidative capacities of mice affected by HF is preserved by 8 weeks of 3VitB cocktail treatment. (A) Immunoblotting of PGC-1α, total-P53 (P53) and acetylated P53 (P53-acetyl). (B) Immunoblotting of total AMPK (tAMPK), phosphorylated-AMPK (AMPK-P), total ACC (tACC)/phosphorylated-ACC (ACC-P)

While the increase in the expression of PGC-1α downstream cascade genes in male and female TAC-3VitB groups (Fig. 6A) could explain the preservation of mitochondrial oxidative capacities (Fig. 5A), the absence of any variation in PGC-1α gene expression and protein level between the different experimental groups suggested a post-translational activation of this transcriptional coactivator. Amongst the strongest activators of PGC-1α, SIRT1 requires NAD^+^ for its deacetylase activity which is thus largely dependent on NAD metabolism. 3VitB cocktail prevented the significant drop of NAD level induced by TAC in males while NAD level was not altered by TAC in females (Fig. 5F). Whereas the vitamin treatment could help maintain NAD level, a prerequisite for optimal SIRT1 activity, this deacetylase did not show any significant changes in protein level in the different groups in both sexes, even though a trend towards a higher SIRT1 content was noticed in females after treatment (Fig. 7A). The acetylation level of P53, a target of SIRT1, tended to increase in both TAC-ND groups and was significantly reduced after 8 weeks of vitamins treatment in females only, revealing the activation of SIRT1 by the 3VitB cocktail in females (Fig. 7A). The absence of p53 deacetylation in males suggested that another energy signaling pathway is preferentially activated. As we previously showed that mitochondrial functions in males are especially sensitive to AMP-activated protein kinase (AMPK) while this pathway is dispensable in females [21], activation of this kinase was assessed by measuring phosphorylated-AMPK (P-AMPK) and phosphorylated-acetyl-CoA carboxylase (P-ACC), a major AMPK target used to reveal AMPK activation. P-AMPK/total-AMPK ratio was unchanged in all male groups, but TAC-3VitB group exhibited significantly higher P-ACC/total-ACC ratio after 8 weeks of 3VitB treatment, unveiling the activation of AMPK in this group (Fig. 7B). In contrast, AMPK phosphorylation level was repressed by TAC in females and no activation by the 3VitB cocktail was noticed as measured by ACC phosphorylation level (Fig. 7B).

## Discussion

These last years, intensive research led in the field of HF has allowed a better understanding of the physiopathology of this syndrome. Although grey areas remain, the research community agrees that energy metabolism alterations in the myocardium plays important roles in cardiac decompensation and should be a target of future therapies [3, 22]. Despite this, so far, no metabolic therapy of HF is routinely used in clinics to specifically improve failing myocardium energy metabolism, especially mitochondrial functions [23]. Based on the protective action of three B vitamins (NR, folate and cobalamin) on energy metabolism of the heart in animals facing cardiac pressure overload when administrated separately and preventively in our previous studies [10, 11], we tested the potential positive effects of a cocktail of these vitamins (3VitB) in a murine model of established HF to investigate its curative use. Here, we showed that the introduction of a diet supplemented with B3, B9 and B12 in mice with established cardiac dysfunction exhibits beneficial effects with stronger impacts in males although numerous beneficial effects were also seen in females. Precisely, this study shows that this combination of three vitamins slows down the degradation of cardiac function induced by pressure overload, improves the physical capacities of the animals and protects myocardium energy metabolism, especially mitochondrial oxidative capacities; these effects are more pronounced in males with significant improvement of survival in these mice that are more severely affected by TAC in comparison with females.

In recent years, the studies aiming at understanding the metabolic alterations encountered in HF demonstrated that structural and functional alterations in mitochondria are associated with major deregulations of NAD homeostasis [4, 24]. Beyond the fact that NAD as a coenzyme is required to support fuel substrate oxidation, TCA cycle and oxidative phosphorylation in mitochondria to generate ATP, loss of NAD homeostasis has also been reported to impact enzymes with NAD^+^-dependent activity, such as Sirtuins, that could be involved in cardiac disease progression [25–27]. It was therefore with the idea of proposing a treatment that could both stimulate the fundamental processes of energy metabolism and support cellular levels of NAD that the combination of B vitamins tested in this study was developed, hypothesizing that the addition of the respective effects of these vitamins on mitochondrial biogenesis and NAD metabolism [10, 11] could constitute a new tool in the therapeutic arsenal for treating HF. While the significant reduction in mortality in males is a strong argument in favor of the efficacy of this B vitamin cocktail in slowing the progression of the disease, the improvement in physical capacities observed in both sexes is also highly significant, since it demonstrates better adaptation of the treated animals when the body’s energy demand increases. By extrapolation, this would be akin to a better exercise test score in HF patients, a predominant indicator in determining the stage of HF [28]. Moreover, as exercise training and cardiac rehabilitation are considered as major non-pharmaceutical beneficial approaches in the treatment of HF [29], the increased physical capacities conferred by the 3VitB cocktail could enhance the benefit of these approaches. Thus, the subtler effects of this treatment on mortality and cardiac systolic function in the females, resulting from a milder phenotype that was expected due to widely-reported cardioprotective mechanisms in this sex [30], should not obscure its benefits in female mice.

Incidentally, the improvement in diastolic function, the reduction in pathological β−MHC isoform expression, and the milder cardiomyocytes hypertrophy in TAC females after treatment, comparable to what was observed in males, demonstrates the sensitivity of the females to these vitamins. Interestingly, 3VitB treatment improved physical capacities in exercise stress test in females despite a lack of clear improvement of systolic cardiac function although the latter was less affected than males. Moreover, we note that diastolic dysfunction as assessed by E/A was improved in females as in males. Obviously, physical exercise capacities integrate a number of regulatory physiological processes and we measured the cardiac function parameters at rest since limitation of the murine model precludes to perform echocardiography while exercising. Therefore, we cannot know if the 3VitB treatment improves cardiac adaptation to exercise or other parameters as well including vascular resistance as reported for NR [31], skeletal muscle bioenergetics or respiratory function, as suggested by the reduced level of pulmonary oedema obtained with the 3VitB treatment.

**Fig. 8:**
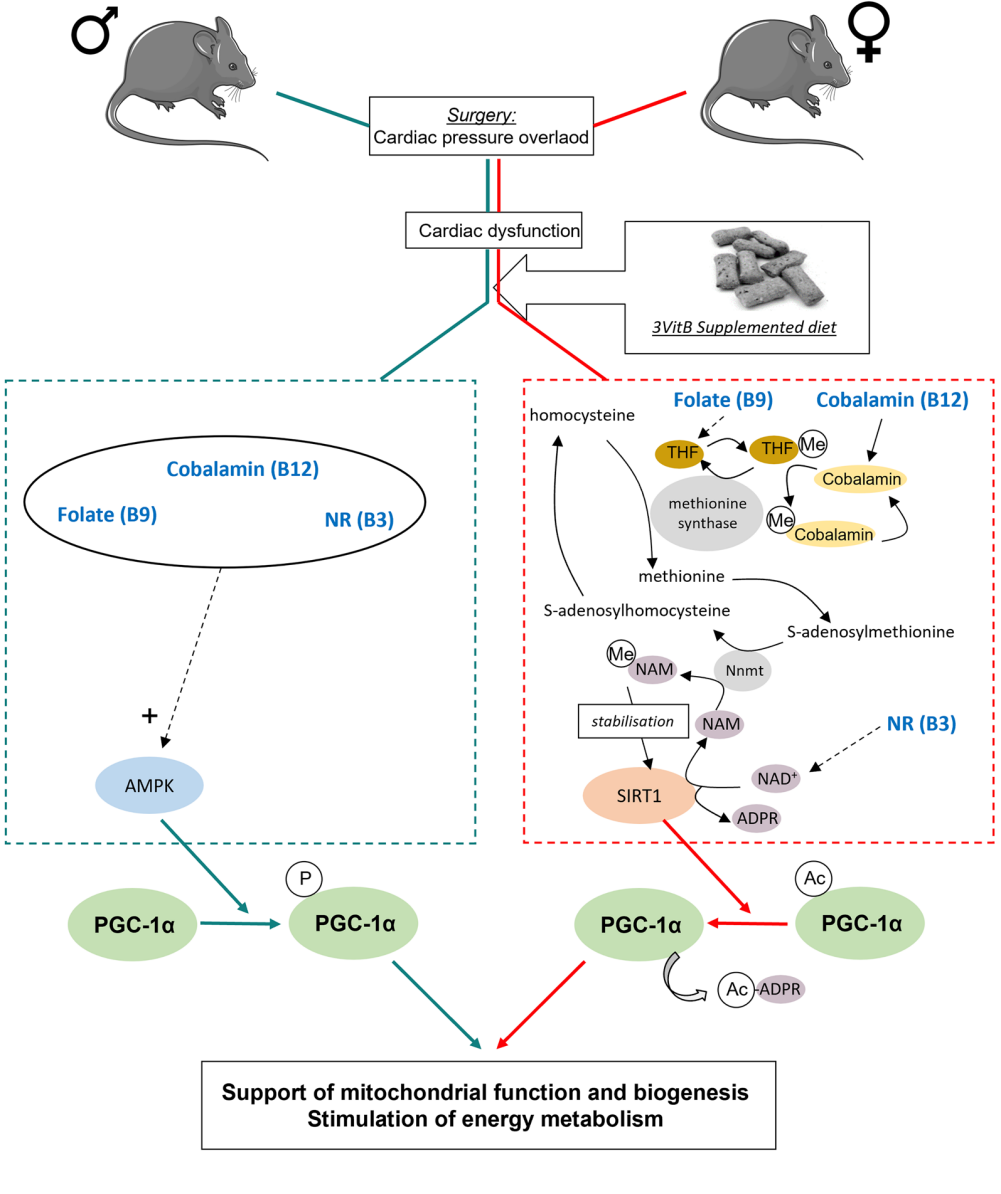
Potential molecular mechanisms activated by 3VitB treatment to support cardiac energy metabolism. In males, the effect of the B vitamins on cardiac metabolism and function would involve AMPK that could be activated by the 3VitB cocktail through pathways that remain to be explored. This activation of AMPK would induce a stimulation of PGC-1α through its phosphorylation. This would stimulate mitochondrial biogenesis and diverse facets of cardiac energy metabolism, thereby helping the heart cope with pressure overload. In females, 3VitB treatment activates SIRT1 that could be a key element in the beneficial effects of these vitamins. The support of SIRT1 activity in this model could be due to a synergistic action of the three vitamins, leading to the stabilization of SIRT1 which would deacetylate and activate PGC-1α with subsequent effects on mitochondrial biogenesis and cardiac energy metabolism. (Ac, acetyl moiety; ADPR, adenosine diphosphate ribose; Me, methyl moiety; Nnmt, nicotinamide N-methl-transferase; THF, tetrahydrofolate)

In the cardiac left ventricle, the study of the mitochondrial respiratory chain on permeabilized ventricular fibers clearly highlighted an improvement in the oxidative capacity of mitochondria in treated males and females. In the latter, these improved oxidative capacities are not associated with any significant effects of the vitamins on citrate synthase enzyme activity, a marker of functional mitochondrial mass, that remains degraded in 3VitB treated females compared to sham females. This was not the case in males, where the 3VitB cocktail normalized the activity of this enzyme, suggesting again that the treatment acts through different pathways according to sex. However, the activation of the expression of genes that are under the control of PGC-1α such as *Tfam*, *Cox4* and *Mcad* [32], shows that the support of mitochondrial oxidative capacities, involves mechanisms that stimulate genes involved in energy metabolism in both sexes. It is now well known that PGC-1α plays a central role in the regulation of energy metabolism and that its expression and activity can be modulated through many ways [33]. The various mechanisms leading to the activation of this major regulator of energy metabolism and mitochondrial life cycle in particular could therefore be differently stimulated by the B vitamins in males and females in the present model (Fig. 8). These mechanisms could involve SIRT1, which appears to be activated only in treated females. SIRT1 is a deacetylase whose actions on PGC-1α activity and on metabolism in general has been highlighted in several studies [17, 34, 35], and it could therefore be at the heart of mechanisms protecting energy metabolism. Deregulations of pathways involving SIRT1 have been described in the pathophysiology of HF [36], and the effects of a combination of cobalamin and folate [10, 37] and NR [38] on the activity of this enzyme could play a crucial role in the benefits observed in females. In males, SIRT1 does not seem to be activated, suggesting other pathways turn on to mediate the protective effect of the 3VitB therapy. Among these pathways, the stimulation of AMPK revealed in males in the present study could play a role in improving the survival of the animals included in this study. AMPK, a key metabolic sensor, is known for its cardioprotective effects [39–41] and it has already been shown that AMPK phosphorylation or/and activity is increased under NR or Cobalamin-folate treatment in several tissues/cell types, although the underlying mechanisms have not been identified [10, 42, 43]. We showed that AMPK controls the regulatory mechanisms of energy metabolism in a sex-specific manner, being essential in males to reduce cardiac fibrosis and maintain mitochondrial function and structure but dispensable in females [21]. Its activation only in males in the present study could thus partly explain the differences observed between both sexes, its involvement in the sex-specific effects of the 3VitB cocktail will thus be the subject of a future work. Interestingly, the differences in the response between males and females could also involve complex interactions with other components of the diet. Indeed, in a recently published collaborative study by our teams [44], this same 3VitB cocktail was mixed with a synthetic diet whose composition (amino acids, fat, carbohydrates, minerals and vitamins) matching the composition of the natural diet that we used in the present study. The 3VitB synthetic diet was provided to mice of the same genetic substrain C57BL6N, starting 4 weeks after TAC as the present study. Interestingly, mixing the 3VitB cocktail with the synthetic diet blunted the benefit of the treatment in males but not in females, which, on the contrary, responded positively to the synthetic diet. The 3VitB synthetic diet notably normalized the circulating lipid profile compared to untreated TAC females suggesting improved energy metabolism although mitochondrial function was not measured in this study [44]. Altogether, the results of David et al. [44], and the present study, show that some components in the natural diet that we used in the present study synergize with the 3VitB cocktail to unlock its beneficial action for males and those components are not necessary for the females. We therefore hypothesize that such components could be the phytoestrogens derived from soya that are present in the natural diet and possess estrogen-like activity, and those phytoestrogens would not be necessary in females that have a robust estrogen signaling. An alternative, but not mutually exclusive hypothesis is that differences in the intestinal microbiota between the Canadian [44] and French (present study) mice cohorts contribute to a different response in males and females. Future research will be required to decipher between those complex mechanisms to better understand the interaction between B vitamins and environmental factors. Nevertheless, it’s important to underline that in two independent studies, the supplementation with 3VitB cocktail provides a benefit compared to non-supplemented diet to at least one sex, further supporting the high translational potential of this approach.

A few years ago, the potential beneficial effects of B vitamins, especially cobalamin and folate, in the context of human cardiovascular diseases associated with hyperhomocysteinemia (HHCY) were questioned and led to several clinical trials [45, 46]. HHCY is often due to vitamin B9, and/or B12 deficiency and although the ability of cobalamin and folate to reduce homocysteinemia in patients was confirmed by these studies, the prevention of cardiovascular events by these vitamins was not conclusive enough to recommend treating patients at cardiovascular risk with these compounds. However, these studies only assessed the consequences of the homocysteine-reducing effect of cobalamin and folate on atherothrombotic events in specific cardiovascular diseases; they did not assess vitamin B9 and B12 supplementation in HF patients and they did not take into account the roles of these vitamins in energy metabolism [8] that could be of great value in the context of HF. HHCY has been reported in HF regardless of etiology and is associated with clinical variables [47], and although folate and cobalamin deficiencies seem rare in HF, data suggest that subclinical B12 deficiency may be underestimated in this pathology [48]. Regardless of cause, HHCY may contribute to HF progression since it exerts harmful vascular effects and may directly impair cardiac function (for review see [8]). Therefore, the use of these two vitamins in the treatment of HF to lower hyperhomocysteinemia and stimulate energy metabolism seems to be a sensible strategy, especially since cardiovascular beneficial effects of cobalamin and folate have been revealed in patients older than 69 in Towgifhi’s study after stratifying treated population [49]. Moreover, two meta-analysises of folate supplementation found reductions in cardiovascular events in patients with cardiovascular disease [50, 51]. While these studies give credit to cobalamin and folate as therapeutic agents, the beneficial effects of NR on the cardiovascular system reported by a significant number of preclinical studies have raised hopes for its clinical applications (for review see [8]). Based on evidence of alterations in NAD homeostasis in HF in animal models and HF patients [4, 11], the NAD replenishment strategy has recently led to a clinical trial involving patients with HF with reduced ejection fraction (NCT03423342). This trial demonstrated the safety of NR, which increased total blood NAD levels, improved mitochondrial respiration capacities in peripheral blood mononuclear cells that also expressed less pro-inflammatory cytokines in comparison to placebo [52]. Thus, the use of B vitamins as therapeutic agents to treat cardiovascular diseases seems credible. To date the combination of folate, cobalamin and NR has never been tested in humans in the specific context of HF with reduced ejection fraction, the results produced by the present work demonstrating the support of energy metabolism by the 3VitB cocktail in a model of heart failure induced by pressure overload, are strongly in favor of considering the therapeutic use of these B vitamins in heart failure.

In its advanced stages, HF is a multi-organ syndrome in which systemic inflammation and dysfunction in the kidneys, liver, lungs, endothelium, or skeletal muscle compound the primary cardiac impairment [53]. While our study demonstrated clear effects of 3VitB treatment on cardiac energy metabolism, we cannot exclude the possibility that these vitamins also improved survival and/or physical capacities through actions on other organs or systems. For instance, systemic inflammation, which plays a key role in HF progression, particularly in cardiac fibrosis and coronary microvascular endothelial dysfunction [54], has been shown to be reduced by NR treatment [55, 56]. In a model of sepsis-induced cardiac dysfunction, NAD repletion via NAD precursors lowered circulating proinflammatory cytokines and was associated with reduced damage to the kidneys, liver, and vascular endothelium [57]. Protective effects of NR on the liver and kidneys have also been observed in animal models of hepatic stress and renal stress [58, 59], demonstrating that NR maintains favorable circulating lipid profile and protects renal function from damage commonly seen after myocardial infarction. Obviously, all these actions could be beneficial in the context of our murine model of established HF, could be involved in the positive effects of the 3VitB treatment and could be complementary to the benefits on cardiac energy metabolism reported in the present study.

## Conclusion

In summary, the present work shows, in line with our previous studies, that the oral supplementation with a cocktail mixing folate, cobalamin and NR improves heart function, mitochondrial function and survival in mice facing cardiac pressure overload with a higher sensitivity in males. These vitamins could be an initial tool for targeting metabolic alterations in HF and may be a new option to treat this disease. Despite the considerable work carried out during this study, the precise mechanisms involved in the benefits conferred by this treatment have not been clearly identified, and this remains a major limitation of our study. However, AMPK in males and SIRT1 in females could play key roles that will be investigated in future works. Of course, therapeutic use of this compounds will also require further studies to determine pharmacokinetics of these vitamins and to assess safety and efficacity in human before translation to clinics.

## Supplementary Information

Supplementary Material 1

## Data Availability

The datasets used and/or analysed during the current study are available from the corresponding authors on reasonable request.

## Abbreviations

3VitB: Three B vitamins
ACC: Acetyl-CoA carboxylase
ACR: Acceptor control ratio
AMPK: AMP-activated protein kinase
BNP: Brain natriuretic peptide
COX4: Cytochrome c oxidase 4
CS: Citrate synthase
CVD: Cardiovascular disease
HF: Heart failure
HWTL: Heart weight-to-tibia length ratio
LV: Left ventricle
LVDd: Diastolic left ventricular diameter
LVDs: Systolic left ventricular diameter
LVEF: Left Ventricular Ejection fraction
MHC: Myosin heavy chain
NAD: Nicotinamide adenine dinucleotide
NAM: Nicotinamide
NAMPT: Nicotinamide phosphoribosyltransferase
MCAD: Medium-chain acyl-CoA dehydrogenase
NMN: Nicotinamide mononucleotide
NMRK2: Nicotinamide riboside kinase 2
ND: Normal diet
NR: Nicotinamide riboside
NRF1: Nuclear respiratory factor 1
P-ACC: Phosphorylated-acetyl-CoA carboxylase
P-AMPK: Phosphorylated-AMPK-activated protein kinase
PGC-1: Peroxisome proliferator-activated receptor gamma co-activator 1
PRMT1: Protein arginine methyltransferase 1
SAM: S-adenosine methionine
SIRT1: Sirtuin-1
SV: Stroke volume
SOD2: Superoxide dismutase 2
TAC: Transverse aortic constriction
TFAM: Mitochondrial transcription factor A
