# Supplementary material for: Sex-specific benefits of a combined supplementation of B vitamins, nicotinamide riboside, folate and cobalamin, in a murine model of heart failure
Source: Biol Sex Differ. 2025 Oct 21;16:82. doi: 10.1186/s13293-025-00764-x (PMC12538762; doi:10.1186/s13293-025-00764-x)
Supplement: Supplementary file 1 — Supplementary Material 1 [file 13293_2025_764_MOESM1_ESM.docx]

**Supplementary information**

**Supplemental methods**

## Pressure overload-induced Heart failure model (TAC)

*Surgery procedure*

The mouse model of heart failure used for this study was a pressure overload induced by a surgical Transverse Aortic Constriction (TAC). Forty-nine-to-fifty-five-day old male and female C57BL/6NCrl mice (Charles River Laboratories, breeding site Italy) were anesthetized by intraperitoneal injection (0.5-mL 29G syringe) of 100 mg/kg ketamine (Imalgene 1000, Merial, France) combined with 10 mg/kg xylazine (Rompun 2%, Bayer healthcare, France) and placed under artificial respiration (MiniVent 845 - Small Animal Ventilator, Harvard Apparatus, France). Mice underwent subcutaneous injection of 1 mg/kg meloxicam (Metacam, Boehringer Ingelheim, Germany) to manage pain during surgery. A subcutaneous injection of 4 mg/kg lidocaine (Lurocaine, Vetoquinol, France) was then given at the site of the incision. The animal was kept on a hot plate (Bioseb, France) at 37°C throughout the surgery and ocular gel (Lubrithal, Centravet, France) was applied to prevent eye drying. Endotracheal intubation was performed to place the mouse under artificial respiration (MiniVent 845 - Small Animal Ventilator, Harvard Apparatus, France) at the frequency of 200 strokes by min for a stroke volume of 200μL. A suprasternal skin incision was made and followed by a mini proximal sternotomy on the upper thorax and the first rib was opened. The thymus was retracted and muscles were dissected to expose the aortic arch. A 7-0 silk suture was passed under the aorta between the brachiocephalic and left common carotid arteries. The transverse aorta constriction (TAC) was done by a ligature around a 27-gauge needle used as a guide on the aorta. After banding, the 27-gauge needle was removed and muscles were stitched. Chest and skin were closed by suture point. Control mice were subjected to an identical procedure without performing ligature (Sham). The animals were rehydrated with physiological saline at 37°C and extubated as soon as they could breathe spontaneously. Upon awakening, each mouse was isolated in a cage in a quiet and warm place. The mice received an injection of 1 mg/kg meloxicam 24 h after surgery and every 24 h for up to 72 h. If the pain followingTAC was intense, an injection of 0.04 mg/kg buprenorphine (Buprécaire, Axience SAS, Pantin, France) was given. Pain management after surgery are detailed in figure S1C.

**Echocardiography**

During echocardiography, mice were placed on a heating plate for small rodents and heart rate, body temperature, and ECG were constantly measured. Cardiac function was evaluated by trans-thoracic echocardiography with a Vevo 3100 device (FUJIFILM Visualsonics Inc., Toronto, Canada) equipped with a linear array 22–55 MHz MicroScan mouse cardiovascular transducer (MS550). Systolic cardiac function parameters such as left ventricular ejection fraction (LVEF), left ventricular shortening fraction (LVSF), left ventricular mass (LV mass), cardiac diameters (left ventricular end-systolic diameter (LVESD) and left ventricular end-diastolic diameter (LVEDD) and volumes (left ventricular end-systolic volume (LVESV) and left ventricular end-diastolic volume (LVEDV), and stroke volume (SV) were obtained by anatomic Bidimentional and M-mode views in parasternal long axis and parasternal short axis. Diastolic cardiac function parameters such as E/A ratio were obtained by apical four-chamber view. Flow velocity, in the aortic arch, was performed using an ultrasound probe MX250 at 15-30 MHz. MX250 probe was selected because, after constriction, the flow velocity is so strong that it is necessary to use a rat probe to visualize the entire peak (more than 4m/s). The animal was placed in a lateral position to visualize the aortic arch and the constriction (in TAC mice). The aortic flow velocity was measured by pulsed wave (PW) Doppler. The flow was disrupted and maximal at the point of constriction. The peaks were measured in triplicate at the point of constriction where the flow is maximally accelerated for each animal (=Velocity Max). Images were analysed by VevoLab Software (FUJIFILM Visualsonics Inc., Toronto, Canada). For converting velocity difference obtained with doppler to pressure gradient, the Bernoulli equation was used. The Bernoulli equation is a difference between aortic velocity and proximal velocity but the simplified Bernoulli equation assumes that proximal velocity can be ignored because it is equal or under to 1m/s. So the pressure gradient estimated using the simplified Bernoulli equation was: (Pressure gradient (Delta P) = 4*(Velocity Max^2)) in m/s.

**Cardiac permeabilized fibers for respirometry assay**

Fibers were dissected from the free wall of freshly harvested left ventricles in S solution (CaK_2_ ethyleneglycol tetraacetic acid (EGTA) (2.77mM), K_2_EGTA [100 nM free Ca2+] (7.23 mM), MgCl_2_ [1 mM free Mg2+] (6.56 mM), Na_2_ATP (5.7 mM), phosphocreatine (15 mM), taurine (20 mM), dithiothreitol (DTT) (0.5 mM), K-methane sulfonate (50 mM), imidazole (20mM), pH 7.1) on ice. Fibers were permeabilized in S solution added with saponin (50µg/ml) during 30 min at 4°C. About 5 mg (wet weight) of permeabilized fibers were placed in each oxygraphic chamber containing respiration solution (CaK_2_ ethyleneglycol tetraacetic acid (EGTA) (2.77mM), K_2_EGTA (7.23 mM), MgCl_2_ (1.38 mM), K_2_HPO_4_ (3mM), taurine (20 mM), dithiothreitol (DTT) (0.5 mM), K-methane sulfonate (90 mM), Na-methane sulfonate (10 mM), imidazole (20mM), 2 mg.ml^-1^ bovine serum albumin, pH 7.1) the temperature of which is monitored at 23 °C.

**Liquid Chromatography-Mass Spectrometry**

*Tissue preparation*

After sampling, heart was reduced to powder and 30 mg were weighed. The extraction was carried out using 800 μl of ice-cold 80% methanol. This is a semiquantitative method based on the use of deuterated nicotinamide (NAM-d4, N407752 Toronto Research Chemicals, Canada), added at the final concentration of 1mM. The tissue was homogenized in the Tissue Lyser (MBIZR-0209-99, MBI Lab Equipment, Canada) at 20 Hz (speed 5 during 45 seconds). After a 15min centrifugation at 16,000 rpm (Sigma 4-16K, MBI Lab Equipment, Canada), the supernatant was collected and evaporated overnight in Speed Vac (RVC 2-33 CDplus, MBI Lab Equipment, Canada) at 5 mbar overnight. The sample was reconstituted with 100 μl of 60% of acetonitrile (A955-4, fisher chemical, Canada) diluted in water. After a sonication step (M1800, Emerson Branson, Canada) and a 2.5 min centrifugation step at 2000 rpm (Sigma 2-7, MBI Lab Equipment, Canada), the sample was transferred into a glass vial and placed in the HPLC coupled with a 6495 triple quadrupole MS/MS system (Agilent Technologies, USA) for the LC-MS analysis.

*LC-MS analysis*

Standards and samples (6µl) were injected onto Agilent InfinityLab Poroshell 120 HILIC-Z column, 2.1 mm × 150 mm, 2.7 μm, PEEK-lined combined with UHPLC Guard Infinity Lab Poroshell HILIC-Z 2.1 x 5 mm, 2,7m (Agilent 821725-947). For the high pH method, mobile phase A was ammonium acetate [10 mM, pH 9] adjusted with ammonium hydroxide. Mobile phase B was composed of 85% of acetonitrile combined with ammonium acetate [10 mM, pH 9] adjusted with ammonium hydroxide with a flow rate of 250 μl/min at a controlled temperature of 25°C. To minimize the binding of polar ionic metabolites to trace levels of metal in the system, InfinityLab Deactivator Additive (p/n 5191-4506, Agilent Technologies, USA) was added to the high‑pH mobile phase. The analysis was done in positive mode. The following linear gradient was run for 20 min (0–3min 98% B, at 11 min 30% B, 12min 40% B, 16-18 min 95%B, 18-20min 2% B). To determined retention times of Methyl-NAM, a standard molecule was used (1-Methylnicotinamide chloride (MeNAM) Sigma M4627). Retention times are shown in Table S2. Relative semi-quantitative values were determined using area ratios of the internal standard NAM-d4. Data were processed and analyzed with the Mass Hunter Qualitative Analysis (Agilent Technologies, version B.06.00). The reproducibility of the semi-quantification method was evaluated by calculating inter- and intra- assay coefficients of variability (CV) in 30 mg of control dog cardiac tissue powder (Table S3).

**Supplemental figure legends**

**Figure S1: Schematic experimental design of the study divided into two main batches.**

**A.** Evaluation of 3VitB cocktail impacts on survival and cardiac function at 20 weeks of treatment. The randomization was done at 4 weeks after TAC surgery based on three inclusion criteria (pression gradient, LVEF, and LV mass). Males and females were divided into three groups: 1) Sham: sham-operated mice receiving standard non-synthetic diet (n=11 males and n=11 females), 2) TAC-ND: TAC mice receiving standard non-synthetic diet (n=20 males and n=16 females), 3) TAC-3VitB: TAC mice receiving supplemented diet with NR, cobalamin and folate (n=19 males and n=20 females). Cardiac function was monitored by echocardiography every four weeks.

**B.** Evaluation of 3VitB cocktail impacts on cardiac tissue, HF and fibrosis biomarkers, mitochondrial functions, NAD metabolomic and effort tolerance at 8 weeks of treatment. The randomization was done at 4 weeks after TAC surgery. Males and females were divided into three groups: 1) Sham: sham-operated mice receiving standard non-synthetic diet (n=9 males and n=6 females), 2) TAC-ND: TAC mice receiving standard non-synthetic diet (n=11 males and n=10 females), 3) TAC-3VitB: TAC mice receiving supplemented diet with NR, cobalamin and folate (n=11 males and n=10 females).

**C.** Throughout the study, especially for survival monitoring, health status of the animals was assessed using a scoring grid. The animal reaching a total score reaching 5 or displaying a score of 3 for only one of the 3 criteria was euthanized.

**Figure S2: Validation of TAC induced-pressure overload model at four weeks after surgery and mice randomization by echocardiography**

**A.** Four weeks after surgery, the TAC model was confirmed by measuring the aorta diameter by echocardiography trans-thoracic (Vevo 3100, FUJIFILM Visualsonics Inc., Toronto, Canada) in Bidimensional mode (B-mode). The dilation of the aorta before the ligature (site A) and the reduction of the aorta after the ligature (site B) was measured. The view of the aortic cross structure was confirmed by an observation of the aortic flow in color doppler.

**B.** Around the ligature site, the aorta flow was altered and considerably accelerated in TAC model. Pressure gradient was measured by pulsed wave (PW) Doppler for sham and TAC animals using the modified Bernoulli equation (Pressure gradient = 4*Velocity2). Images were analyzed using VevoLab Software (FUJIFILM Visualsonics Inc., Toronto, Canada). Mice with a pressure gradient reaching al least 60mm Hg were included in the study.

**C.** TAC induced-pressure overload model was correlated with an alteration of cardiac function and cardiac hypertrophy. Left ventricle ejection fraction (LVEF) and left-ventricle mass (LV mass) were measured by echocardiography in parasternal long and short axis B-mode and M-mode to refine mouse randomization. Only TAC mice dysplaying at least a reduction of 10% of LVEF and 30% increase in LV mass were included in the study.


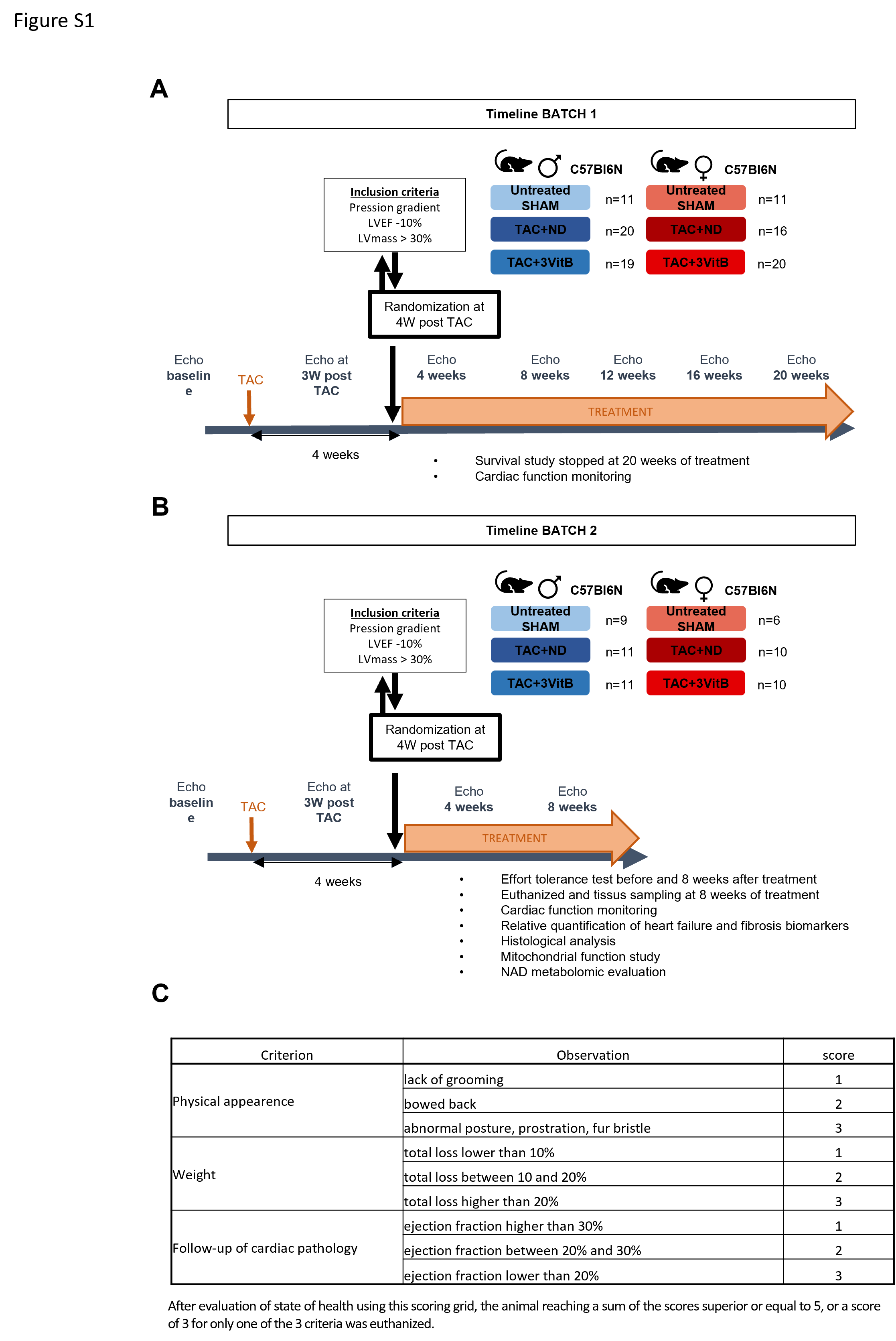


C


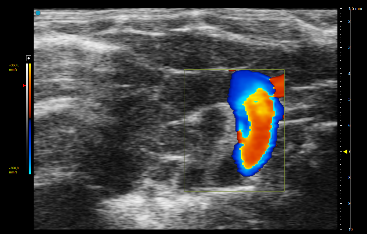

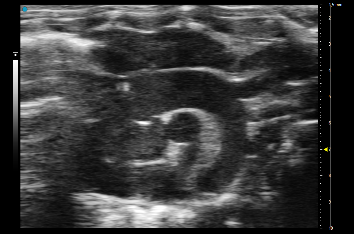

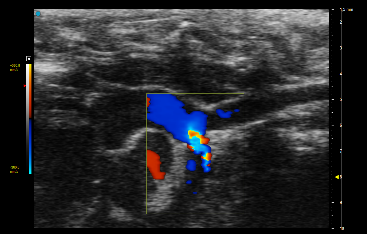

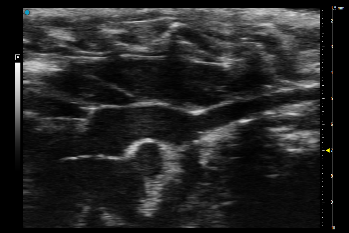


B-Mode

Color Doppler

Sham

TAC

**Male**

**Female**


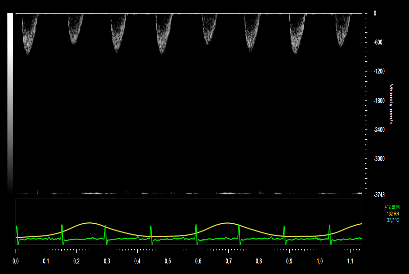

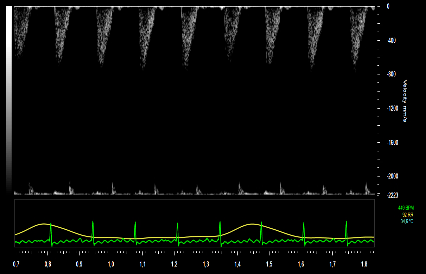

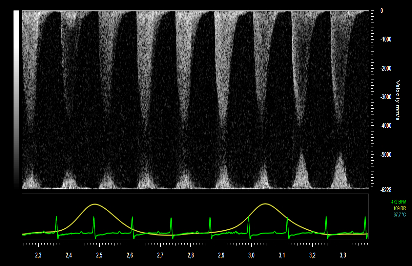

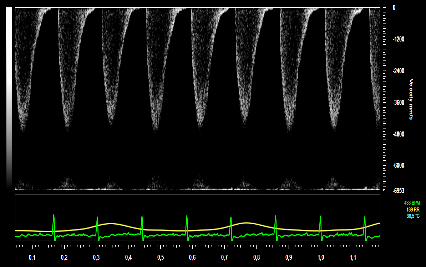


Sham

TAC

A

B

A

B

Pression gradient evaluation

Aortic diameter

Cardiac function

**Sham**

**Sham**

**TAC**

**TAC**

**A**

**B**

**C**

Figure S2

**Table S1.** Primers used for quantification of mRNA expression level.

| **Gene** | **Primers** | **Hybridation temperature** |  |
| --- | --- | --- | --- |
|  |  |  |  |
| *Col1a* | 5'-CTCAAGATGTGCCACTCTGACT-3' | 60°C |  |
|  | 5'-CTCCATGTTGCAGTAGACCTTG-3' |  |  |
| *Col3a* | 5'-GAT GGAAACCCTGGATCAGA -3' | 60°C |  |
|  | 5'-GCACCAGGAGAACCATTTTC-3' |  |  |
| *Cox4* | 5’-TGG GAG TGT TGT GAA GAG TGA -3’ | 57°C |  |
|  | 5’-GCA GTG AAG CCG ATG AAG AAC-3’ |  |  |
| *α-Mhc* | 5'-CCAATGAGTACCGCGTGAA-3' | 58°C |  |
|  | 5'-ACAGTCATGCCGGGATGAT-3' |  |  |
| *β-Mhc* | 5'-ATGTGCCGGACCTTGGAA-3' | 60°C |  |
|  | 5'-CCTCGGGTTAGCTGAGAGATCA-3' |  |  |
| *Mcad* | 5'-CCGTTCCCTCTCATCAAAAG-3' | 60°C |  |
|  | 5'-ACACCCATACGCCAACTCTT-3' |  |  |
| *Nrf2* | 5'-CACTCAACATTTCGGGAAGAG-3' | 60°C |  |
|  | 5'-CTCATTCATCTGTTGCTCTTGG-3' |  |  |
| *Pgc-1α* | 5'-CACCAAACCCACAGAGAACAG-3' | 58°C |  |
|  | 5'-GCAGTTCCAGAGAGTTCCACA-3' |  |  |
| *Pgc-1β* | 5'-TGGAAAGCCCCTGTGAGAGT-3' | 60°C |  |
|  | 5'-TTGTATGGAGGTGTGGTGGG-3' |  |  |
| *Tfam* | 5'-GCTAAACACCCAGATGCAAA-3' | 60°C |  |
|  | 5'-TACTTGCTCACAGCTTCTTTGT-3' |  |  |

**Table S2.** LC-MSMS. Retention time and Method reproducibility validation

| **Compound Name** | **Retention time (min)** | **Polarity** | **Precursor Ion (m/z)** | | **Product Ion (m/z)** | | **Collision Energy(V)** | |
| --- | --- | --- | --- | --- | --- | --- | --- | --- |
| MeNAM | 7.15 | + | 137 | | 78 | | 20 | |
| MeNAM | 7.15 | + | 137 | | 94 | | 20 | |
| NAM-d4 (internal standard) | 1.701 | + | 127 | | 84.1 | | 20 | |
| NAM-d4 (internal standard) | 1.701 | + | 127 | | 56.1 | | 30 | |
|  |  |  |  |  |  |  |  |  |
| *Reproductibility validation* |  |  |  |  |  |  |  |  |
| **Compound name** | **Transition** | **internal standard** | **inter** | | | **intra** | | |
|  |  |  | **ratio mean** | **ratio STD** | **CV (%)** | **ratio mean** | **ratio STD** | **CV (%)** |
| MeNAM | 137.0 -> 94.0 | NAM-d4 | 0,06645 | 0,00559 | 8,41859 | 0,0831 | 0,01452 | 17,47226 |

**Table S3.** Statistical analysis

|  |  |  |  | Source of Variation | | | Males - Females | | |
| --- | --- | --- | --- | --- | --- | --- | --- | --- | --- |
|  |  |  |  | Interaction | Sex | Treatment | Sham | TAC-ND | TAC-3VitB |
| fig 1 | An. Probability of survival | | P value | 0,47 | 0,24 | <0,001 | ns | * | ns |
|  | C. Mean body weight variation per day | | P value | 0,04 | 0,02 | 0,004 | 0,01 | 0,56 | 0,07 |
|  | D. Exhausting time (Before) | | P value | 0,56 | 0,02 | <0,001 | 0,04 | 0,24 | 0,39 |
|  | D. Exhausting time (After) | | P value | 0,57 | <0,001 | <0,001 | 0,1 | 0,1 | 0,003 |
| fig 2 | A. Mean EF variation per day | | P value | 0,06 | 0,69 | <0,001 | 0,69 | 0,12 | 0,07 |
|  | B. Mean SV variation per day | | P value | 0,16 | 0,5 | 0,002 | 0,75 | 0,72 | 0,04 |
|  | C.Mean LVDs variation per day | | P value | 0,12 | >0,99 | 0,005 | 0,96 | 0,13 | 0,16 |
|  | D.Mean LVDd variation per day | | P value | 0,5 | 0,01 | <0,001 | 0,59 | 0,01 | 0,11 |
|  | E. Ratio E/A | | P value | 0,25 | 0,9 | <0,001 | 0,53 | 0,17 | 0,47 |
|  | F. mRNA 𝛃Mhc | | P value | 0,1 | 0,12 | 0,01 | >0.99 | 0,007 | 0,91 |
|  | F. mRNA 𝛂Mhc | | P value | 0,75 | 0,93 | 0,006 | >0.99 | 0,64 | 0,55 |
|  | F. mRNA 𝛃/𝛂 Mhc ratio | | P value | 0,19 | 0,17 | 0,01 | >0.99 | 0,02 | 0,87 |
|  | F. mRNA Bnp | | P value | 0,003 | 0,05 | <0.001 | >0.99 | <0.001 | 0,57 |
| fig 3 | A. Heart weight/tibia length | | P value | 0,04 | <0,001 | <0,001 | 0,02 | <0,001 | 0,08 |
|  | B. Lung weight/tibia length | | P value | 0,34 | 0,03 | <0,001 | 0,06 | 0,83 | 0,11 |
|  | D. Cardiac fibers distribution 100 | | P value | 0,12 | 0,16 | 0,002 | 0,07 | 0,11 | 0,39 |
|  | D. Cardiac fibers distribution 100-250 | | P value | 0,74 | 0,62 | <0.001 | 0,82 | 0,81 | 0,43 |
|  | D. cardiac fibers distribution 250-400 | | P value | 0,14 | 0,05 | 0,45 | 0,01 | 0,25 | 0,77 |
|  | D. cardiac fibers distribution 400-550 | | P value | 0,73 | 0,34 | <0.001 | 0,88 | 0,19 | 0,74 |
|  | D. cardiac fibers distribution >550 | | P value | 0,76 | 0,59 | <0.001 | 0,57 | 0,47 | 0,77 |
|  | D. Cross sectional | | P value | >0.99 | 0,83 | <0.001 | 0,88 | 0,88 | 0,94 |
| fig 4 | B. Cardiac fibrosis | | P value | 0,007 | 0,12 | <0.001 | 0,61 | <0.001 | 0,88 |
|  | C. mRNA Collagen type I | | P value | 0,007 | 0,87 | 0,01 | 0,65 | 0,008 | 0,07 |
|  | C. mRNA Collagen type III | | P value | 0,53 | 0,59 | 0,81 | >0.99 | 0,21 | 0,8 |
|  | C. mRNA Collagen type I/III ratio | | P value | 0,08 | 0,65 | 0,02 | >0.99 | 0,04 | 0,25 |
| fig 5 | A. Oxygen consumption with P/M | | P value | 0,61 | 0,96 | 0,01 | 0,47 | 0,75 | 0,53 |
|  | A. Oxygen consumption with Succinate | | P value | 0,65 | 0,98 | 0,006 | 0,47 | 0,7 | 0,67 |
|  | A. Oxygen consumption with amytal | | P value | 0,99 | 0,31 | 0,01 | 0,64 | 0,57 | 0,45 |
|  | A. Oxygen consumption with TMPD | | P value | 0,5 | 0,88 | 0,02 | 0,32 | 0,72 | 0,6 |
|  | A. ACR | | P value | 0,76 | 0,15 | 0,001 | 0,82 | 0,32 | 0,18 |
|  | B. Complex I activity | | P value | 0,004 | 0,29 | <0.001 | 0,03 | 0,01 | 0,07 |
|  | B. Complex IV activity | | P value | 0,88 | 0,08 | 0,12 | 0,51 | 0,26 | 0,17 |
|  | C. Citrate synthase activity | | P value | <0.001 | 0,01 | <0.001 | 0,86 | 0,49 | <0.001 |
|  | C. HADHA activity | | P value | 0,08 | <0.001 | <0.001 | <0.001 | 0,02 | 0,35 |
|  | D. Citrate synthase protein | | P value | 0,37 | 0,54 | <0.001 | >0.99 | 0,14 | 0,67 |
|  | E. SOD2 protein | | P value | 0,38 | 0,06 | <0.001 | >0.99 | 0,08 | 0,13 |
|  | F. myocardial NAD | | P value | 0,11 | 0,06 | 0,08 | 0,89 | 0,008 | 0,5 |
|  | G. myocardial cobalamin (B12) | | P value | 0,26 | 0,17 | <0.001 | 0,63 | 0,86 | 0,04 |
|  | H. myocardial MeNAM (MS/MS) | | P value | 0,03 | 0,08 | <0.001 | 0,66 | 0,77 | 0,002 |
| fig 6 | A. mRNA Pgc-1α | | P value | 0,92 | 0,55 | 0,29 | >0.99 | 0,59 | 0,58 |
|  | A. mRNA Pgc-1β | | P value | 0,41 | 0,23 | 0,06 | >0.99 | 0,74 | 0,07 |
|  | A. mRNA Nrf1 | | P value | 0,47 | 0,81 | 0,15 | >0.99 | 0,51 | 0,29 |
|  | A. mRNA Tfam | | P value | 0,91 | 0,01 | <0.001 | 0,3 | 0,1 | 0,07 |
|  | A. mRNA Cox 4 | | P value | 0,81 | 0,86 | 0,02 | >0.99 | 0,54 | 0,78 |
|  | A. mRNA Mcad | | P value | 0,75 | 0,47 | <0.001 | >0.99 | 0,28 | 0,79 |
|  | B. PGC-1α protein | | P value | 0,42 | 0,09 | 0,36 | >0.99 | 0,26 | 0,07 |
|  | B. NRF1 protein | | P value | 0,23 | 0,1 | 0,004 | 0,64 | 0,08 | 0,14 |
| fig 7 | A. SIRT1 protein | | P value | 0,02 | <0.001 | 0,24 | >0.99 | 0,02 | <0.001 |
|  | A. p53-acetyl protein | | P value | 0,09 | 0,06 | 0,06 | >0.99 | 0,68 | 0,006 |
|  | B. AMPK-P-AMPt | | P value | 0,03 | <0.001 | 0,007 | >0.99 | 0,003 | 0,001 |
|  | B. ACC-P-ACCt | | P value | 0,002 | 0,54 | 0,46 | >0.99 | 0,04 | 0,003 |

|  |  |  |  | Source of Variation | | | Males - Females | |
| --- | --- | --- | --- | --- | --- | --- | --- | --- |
|  |  |  |  | Interaction | Sex | Effet TAC | Sham | TAC |
|  |  |  | 3 weeks post TAC | | | | | |
| fig S2 | A. Aortic diameter | | P value | 0,61 | 0,67 | <0.001 | 0,95 | 0,46 |
|  | B. Pressure gradient | | P value | 0,94 | >0.99 | <0.001 | 0,96 | 0,95 |
|  | C. LV ejection fraction | | P value | 0,6 | 0,67 | <0.001 | 0,59 | 0,91 |
|  | C. LVmass | | P value | 0,13 | 0,15 | <0.001 | 0,97 | 0,006 |
